# Happy Aged People Are All Alike, While Every Unhappy Aged Person Is Unhappy in Its Own Way

**DOI:** 10.1371/journal.pone.0023377

**Published:** 2011-09-08

**Authors:** Michele Tumminello, Salvatore Miccichè, Ligia J. Dominguez, Giovanni Lamura, Maria Gabriella Melchiorre, Mario Barbagallo, Rosario N. Mantegna

**Affiliations:** 1 Department of Social and Decision Sciences, Carnegie Mellon University, Pittsburgh, Pennsylvania, United States of America; 2 Dipartimento di Fisica, Università di Palermo, Palermo, Italy; 3 Department of Internal Medicine and Geriatrics, University of Palermo, Palermo, Italy; 4 Socio Economic Research Centre, National Institute of Health and Science on Aging (I.N.R.C.A.), Ancona, Italy; University of Maribor, Slovenia

## Abstract

Aging of the world's population represents one of the most remarkable success stories of medicine and of humankind, but it is also a source of various challenges. The aim of the collaborative cross-cultural European study of adult well being (ESAW) is to frame the concept of aging successfully within a causal model that embraces physical health and functional status, cognitive efficacy, material security, social support resources, and life activity. Within the framework of this project, we show here that the degree of heterogeneity among people who view aging in a positive light is significantly lower than the degree of heterogeneity of those who hold a negative perception of aging. We base this conclusion on our analysis of a survey involving 12,478 people aged 50 to 90 from six West European countries. We treat the survey database as a bipartite network in which individual respondents are linked to the actual answers they provide. Taking this perspective allows us to construct a projected network of respondents in which each link indicates a statistically validated similarity of answers profile between the connected respondents, and to identify clusters of individuals independently of demographics. We show that mental and physical well-being are key factors determining a positive perception of aging. We further observe that psychological aspects, like self-esteem and resilience, and the nationality of respondents are relevant aspects to discriminate among participants who indicate positive perception of aging.

## Introduction

Human life expectancy has increased steadily for nearly 200 years and it is expected to continue to do so. While 40 percent of newborns were expected to live beyond age 65 in 1900 in developed countries, it is estimated that if the same pace continues, most babies born since 2000 in developed countries will reach 100 years [1]. During the last century the increase was of about two years every decade; driven in the first decades by improvements in sanitation, housing and education, with a steady decline in early and mid-life mortality mostly due to infections. The increased life expectancy in the latter decades was attributable to a decline in late-life mortality, related to medical advances such as the treatment of hypertension and ischemic heart disease, and improved socioeconomic conditions [2]. The remarkable effect of societal conditions was shown by the rapid rise in lifespan in former East Germany after the fall of the Berlin's Wall [3]. Worldwide the number of persons aged 60 and over is anticipated to rise from its current 740 million to reach 1 billion by the end of this decade, and possibly 2 billion by mid-century. There is little doubt that aging of the populations is changing profoundly the societies around the world [4], especially due to key challenges such as those concerning the economic and financial impact of the retirement and care of a growing number of older persons. This will place demands on expensive retirement and care systems while there will be relatively fewer young workers to meet a range of economic demands. This also implies a declining availability of caregivers facing an increasing demand, owing to a range of factors, including an increased rate of women employment, a rising trend of isolated households, and an increasing life expectancy of disabled elders, among others. The recent economic crisis only augments the pressure on these challenges. Hence, the expected demographic trends centre the attention on the importance of health, fulfillment, independence, dignity, and participation of older persons in the society.

The imminent rapid increase in numbers of aging adults will also impact the health systems in the coming years. The oldest-old group (older than 85 years), which is the most rapidly growing segment of the population in developed countries, is the most susceptible to disease and disability [5]. Indeed, population aging has revealed the limits in the management of growing old communities with multiple co-morbidities. Hence, the search for ways to prolong health expectancy with effective prevention of disability has become a primary goal in medicine. The imperative is to allow older adults to maintain their independence and health for as long as possible preventing the harm of chronic diseases and disabilities [2], [6]. Even if disability decreased in past decades in older populations in the US [7] and Europe [8], it still remains remarkably high. Furthermore, recent data suggest that disability rates did not change between 2000 and 2005 among older non-institutionalized Americans [9]. The cost of care for disabled population is about ten fold that of non-disabled people [7]; hence, there are not only important humanitarian, but also economic reasons to improve quality of life during aging.

Aging populations exhibit high degree of heterogeneity and diversity [2], [10], [11]. Heterogeneity refers to variability within the individual while aging, and diversity refers to the position of different groups in relation to one another within society. In order to promote aging well among adults, policies and environment support are needed that enhance lifestyle choices for successful, productive, healthy aging [4]. The perception of aging as a negative event should be discouraged and the determinants of factors that influence the (self)perception of aging need to be studied. In this paper we address this issue by investigating the results of a survey of 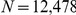 people aged 50 to 90, from 6 West European countries, in the context of the collaborative cross-cultural “European study of adult well being” (ESAW). The project aimed to develop a globally applicable model of “Aging Well”, estimating the direct causal contribution of five key components, personal characteristics and culture, to the outcome variable “Aging Well”: (i) physical health and functional status, (ii) self-resources, (iii) material security, (iv) social support resources, and (v) life activity. Six West European countries participated in the study: Austria (AT - 2,111 respondents), Italy (IT - 2,018), Luxembourg (LU - 2,145), the Netherlands (NL - 1,934), Sweden (SE - 2,417) and the United Kingdom (GB - 1,853). The six-nation European Study ESAW represents a regional component of the Global Ageing Initiative (GAI) involving Australia, Austria, UK/Wales, Greece, Malta, Italy, Sweden, Ghana, China, Netherlands, Luxembourg, Canada, USA, India, Costa Rica, with additional countries seeking involvement - see www.indiana.edu/~caa for information regarding the GAI research project. ESAW is a comparative, cross-cultural study of aging, and has the objective to discover the degree to which different explanatory models of “aging well” fit within participating European nations. In the ESAW study the concept of aging well is indeed studied by describing the unique position of each of the ESAW nations as a part of the fifteen EU-nations (1999: Austria, Belgium, Denmark, Finland, France, Germany, Greece, Ireland, Italy, Luxembourg, the Netherlands, Portugal, Spain, Sweden and the United Kingdom), before the European Union enlargement from Eu15 to Eu25 (2004) and then to Eu27 (2007). Therefore, the six nations (ranging from Scandinavia to the Mediterranean) involved in ESAW are belonging to a subset of nations within the EU on 2002, with a comparable situation with respect to aging well.

In its original form, the database comprehends all the answers given by respondents to a 504-item questionnaire divided in 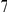 sections. Section A provides background information about the respondents, e.g. home country, age, education, living in a city or in countryside. A summary of demographic details about the respondents is given in Ref. [12]. Section B deals with social support resources. Section C provides information about physical health and functional status. Section D aims to evaluate the mental efficacy. Section E considers life activity, while section F material security. Finally, section G evaluates the self-perception of aging. The English version of the questionnaire is provided in the data S1.

The database is characterized by a pronounced variety in terms of possible answers to each question. There are questions allowing only two answers (e.g. sex) and questions with over 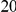 possible answers. We will discuss this variety in the next section. The original structure of the database has been modified by us, in order to remove possible sources of statistical bias influencing the analyses. The modification process aggregating some answers or including them into categorical variables was carried out in three successive steps. In the first step, we aggregated together questions nested in cascade to obtain a list of formally independent questions. In the second step, we removed from the database all the 64 questions to which at least 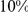 of respondents refused to answer. The third step consisted in removing section E from the analyses because of the different structure of questions in this section, describing life activities that might be specific to each respondent. All details about the database modification are provided in the text S1. After the modification process, there were 258 questions remaining, all of them having answers in a form of categorical variables. The total number of possible answers after the modification process of the database is 1344.

We investigate the modified database by considering the survey as a bipartite network in which individual respondents are linked to the actual answers they provide. This approach allows us to investigate the similarity between profiles of individual respondents and to classify them in communities, which are obtained independently of demographic parameters. Many complex systems present an intrinsic bipartite structure, and are often described and modeled in terms of networks [13]–[17]. Examples include movies and actors [13], [14], [16], authors and scientific papers [18]–[21], email accounts and emails [22], plants and animals that pollinate them [23], [24]. In the present study, we look at the survey as a bipartite system in which on one side are the respondents to the survey and on the other side the complete list of answers in the questionnaire. A bipartite network is therefore obtained by linking each respondent with all the answers she/he actually gives. This description of the survey as a bipartite network allows us to construct a statistically validated network of respondents, in which each link represents a statistically validated similarity between the survey profiles of the connected respondents.

## Methods

In this section, we describe the method we use to construct a statistically validated network of respondents to the survey. Each link of the network corresponds to a statistically validated similarity between the survey profiles of linked respondents. The statistical validation is performed against a suitable null hypothesis, which takes into account both the different profile of respondents and the different nature of answers. The general method to construct a statistically validated network in bipartite complex systems has been introduced by some of the authors in a recent paper [25]. As mentioned, we propose to investigate a survey as a bipartite network in which elements of a set correspond to respondents, while elements of the other set include all the answers to questions in the survey. It is to notice that in the present survey each respondent gives one and only one answer to each question. “Not answered” is indeed included in the list of possible answers to a given question. These constraints require to consider a suitable null hypothesis to statistically validate the similarity between two profiles of answers.

### Statistically validated network of respondents

**Figure 1:**
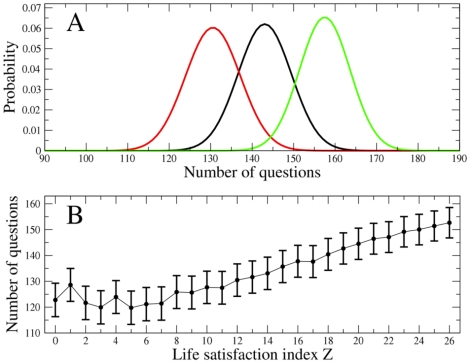
Homogeneity of profile of various groups of participants to the survey. Panel A shows the probability mass function 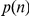 (black line), 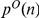 (red line), and 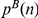 (green line). These probability functions are estimated excluding demographics (section A) and measure the degree of homogeneity of all 12,478 respondents, of the 5,658 respondents in group 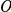 and of the 6,820 respondents in group 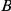, respectively. Panel A shows that the homogeneity of participants involved in the Bonferroni network (group 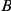) is larger than the homogeneity of all participants, which in turn is larger than the homogeneity of participants excluded from the Bonferroni network (group 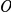). Panel B compares the homogeneity of groups of participants characterized by the same life satisfaction index Z (x-axis). The number of respondents in each group is: Z = 0 (26 participants), Z = 1 (17), Z = 2 (72), Z = 3 (68), Z = 4 (92), Z = 5 (123), Z = 6 (167), Z = 7 (142), Z = 8 (254), Z = 9 (236), Z = 10 (353), Z = 11 (309), Z = 12 (524), Z = 13 (405), Z = 14 (608), Z = 15 (485), Z = 16 (758), Z = 17 (663), Z = 18 (924), Z = 19 (775), Z = 20 (1062), Z = 21 (768), Z = 22 (1077), Z = 23 (663), Z = 24 (821), Z = 25 (318), Z = 26 (361). In the y-axis, we report the average of the probability mass function 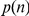 calculated separately for each group of participants by excluding section A of the questionnaire and all questions 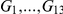. Error bars correspond to the standard deviation of the distributions. Panel B shows that the homogeneity of participants increases with Z.

Let us consider a question 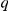, and indicate the 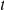 possible answers to this question with 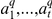. We estimate the probability 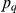 that two randomly picked respondents to the survey answer the same to question 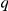 as:
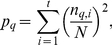
(1)where 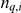 is the actual number of respondents who give the answer 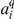 to question 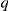. We associate a variable 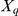 following the Bernoulli distribution 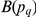 with each question 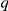 of the survey, and focus on the random variable 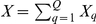. Here 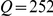, which corresponds to the total number of selected questions (258) minus 6, which is the number of questions related to demographics (section A of the questionnaire). We did not consider section A of the questionnaire to quantify the similarity between respondents' patterns, in order to avoid the influence of demographics on that measure. The variable 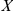 is therefore the sum of 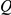 Bernoulli distributed random variables. The probability 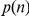 is the probability that two randomly selected respondents answer the same to 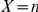 questions. We use an iterative procedure [26] to numerically, and exactly, estimate the probability 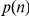. In Fig. 1A, we show the probability mass function 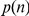 (black line). The probability 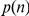 quantifies the similarity of respondents according to their answers to the questionnaire. This quantity does not take into account the presence of other sources of similarity between respondent profiles than the average trend. We therefore use 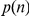 as null hypothesis against which to test the actual number 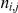 of identical answers as given by each pair 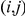 of respondents, i.e. we calculate the 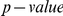 associated with 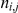 according to the distribution 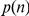: 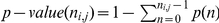. We construct the network of respondents by setting a link between respondent 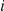 and 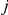 if the p-value associated with 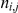 is smaller than the statistical threshold 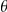. The total number of respondent pairs is 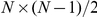. We are testing all the pairs simultaneously and we are therefore performing a multiple hypotheses test [27], which requires one to correct the value of 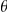 for multiple comparisons. In the present study we use the most stringent correction, which is the Bonferroni correction [27]. We accordingly set 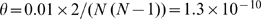, and we indicate the network of respondents obtained with this value of the statistical threshold as the Bonferroni network [25]. The Bonferroni correction is the most restrictive multiple hypothesis test correction used in statistics. In principle, it is possible to consider a less restrictive correction for multiple comparisons, such as the false discovery rate (FDR) correction [25], that gives rise to a network with more links and vertices. Here we decide to investigate only the Bonferroni network because it is highly informative about the system and it is ensuring the lowest level of false positive links. Indeed the probability that only one link of the Bonferroni network is a false positive is smaller than 1%.

### Data collection and ethics statement

ESAW project was a multinational research project involving 6 different countries (see the home page of the project for details: http://esaw.bangor.ac.uk1/index.htm). Two of the authors of the present paper - Giovanni Lamura and Maria Gabriella Melchiorre - were part of the project. Specifically, Giovanni Lamura was the Principal Investigator for Italy (see http://esaw.bangor.ac.uk/research_team.htm). Mario Barbagallo and Ligia J. Dominguez also participated to the project leading the subunit responsible for the major Italian islands. The Italian team of the project collected data of Italian respondents (2,018 over a total of 12,478).

The protocol set by the ESAW project stated that “Principles expressed in the Helsinki Declaration and the conventions of the Council of Europe on Human Rights would be complied with”. However, the survey did not consider any clinical tests to be performed for the respondents. In fact it was “based on individual interviews administered by means of a structured questionnaire to national samples of 1,800–2,500 non institutionalized subjects (e.g. not hospitalized nor in long term care facilities), aged 50–90, in each of the involved countries” (see the ESAW home page of the web site). For this reason an IRB approval was not requested, although “the partners in ESAW have considered the ethical aspects of this project. In fact, ESAW partners have agreed to adhere to a statement of principles and collaborative research agreement. In July 1999 all partners signed the document: Statement of Principles and Collaborative Research Agreement (including data ownership and data sharing, authorship principles, research ethics and responsibilities).”

Concerning the informed consent, they were both written and oral depending on the corresponding National law. In the case of Italy the informed consent was obtained in written form. In all cases, the protocol of the project stated that: “subjects would be reassured that all information gathered in the course of the study would be confidential and that no personal identification information will be released at any time”. The project funders did not require clinical ethical approval to be obtained [28].

## Results and Discussion

### Characterization of respondents in the Bonferroni network

The total number of respondents who show at least one validated link is 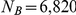. The remaining 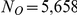 respondents are outside the statistical validated network, at the chosen level of statistical significance. We can therefore split the respondents in two groups, namely the group 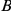, which is formed by the 6,820 respondents involved in the Bonferroni network, and the group 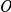 including the 5,658 isolated respondents. These two groups are rather different in terms of the homogeneity of their answers. In Fig. 1A we report the probability 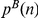 (green line) associated with the group 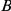, and the probability 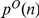 (red line) associated with group 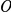. These probabilities are calculated in exactly the same way as 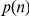 is calculated, but restricting the evaluation in Eq. (1) to the group 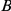 of respondents, in order to estimate 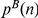, and to the group 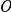 of respondents, in order to obtain 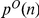. It is interesting to note that the two curves are very different. The mean of 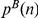 is 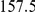 and the standard deviation is 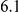, while the mean of 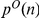 is 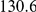 and its standard deviation 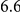. Therefore respondents involved in the Bonferroni network are much more homogeneous, according to the survey, than isolated respondents. We also use the survey to characterize respondents belonging to each of the two groups 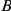 and 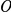 [29]. Let us consider a specific answer 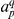 to question 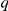, and indicate the actual number of people who give that answer as 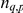. If we assume that groups 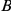 and 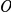 are formed by randomly splitting the 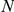 respondents in two groups of size 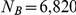 and 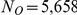, respectively, then the number of respondents 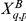 (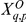) in group 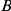 (group 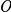) who give the answer 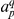 to question 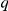 follows the hypergeometric distribution. Specifically, the probability 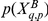 that 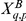 people from group 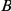 give the answer 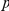 to the question 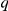 is given by:
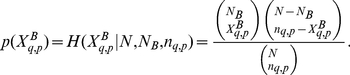
(2)Similarly, we have
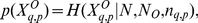
(3)for the group 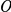. Eq.s (2) and (3) allow one to associate a *p*-value with the observed number of people 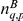 (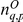) from group 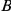 (group 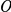) who give the answer 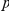 to question 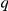. This is directly obtained from Eq. (2) and Eq. (3) as the probability that 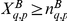 for the group 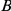, and as the probability that 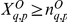 for the group 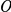. We calculate the p-value for each answer to each question. In Table 1 we show the results of this analysis concerning the section G of the questionnaire. Section G asks respondents about their satisfaction with life. Respondents are asked to indicate whether they *agree* or *disagree* with 13 statements. They are also allowed to answer *not sure* about their feeling with the statement and to pass the statement over (*NA*). The answer 2 is given when the agreement (or the disagreement) to a given statement indicates satisfaction with life, the answer 0 when it indicates dissatisfaction, and the answer 1 when the respondent is not sure about her/his feeling with the statement. All of the p-values reported in Table 1 are smaller than 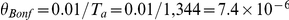, which is the Bonferroni threshold for the present multiple test. Here the total number of tests is 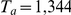. It corresponds to the sum over all the answers of the 258 questions investigated.

**Table 1:** Characterization of groups 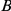 and 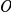 of respondents in terms of life satisfaction.

	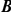 ( 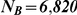 )		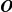 ( 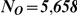 )	
Question	answer	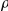	answer	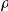
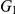	2	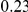	0; 1; 9	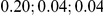
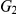	2	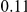	0; 9	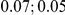
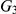	2	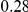	0; 1; 9	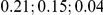
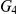	2	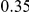	0; 1; 9	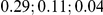
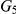	2	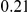	0	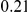
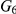	2	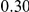	0; 1; 9	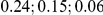
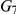	2	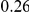	0; 1; 9	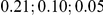
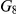	2	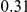	0; 1; 9	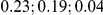
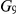	2	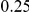	0; 1; 9	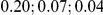
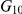	2	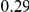	0; 1; 9	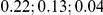
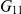	2	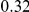	0; 1; 9	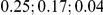
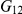	2	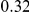	0; 1	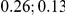
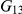	2	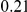	0; 1	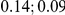

The results summarized in Table 1 show that respondents in group 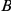 are in general significantly more satisfied with their life than respondents in group 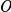. In the Table, we also report the correlation coefficient 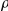 related to the answer to a specific question expressed by the considered group, either 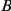 or 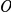. Let us explain how the correlation coefficient is computed by describing a specific example. Let us focus on the group 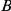 and on a given answer 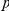 to a given question 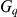. The correlation coefficient 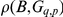 is calculated as the Pearson correlation coefficient between the vectors 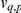 and 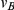. The dimension of both these vectors is 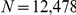, corresponding to the total number of respondents to the survey. The components of the vectors are either 0 or 1. Specifically the components of 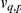 are equal to 1 in correspondence with those respondents who gave an answer 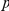 to the statement 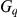, and 0 otherwise. The components of 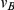 are equal to 1 in correspondence with respondents belonging to group B, and 0 otherwise. In formulae:
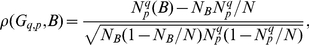
(4)where, 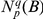 is the total number of respondents in group 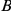 who answered 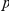 to 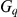, and 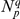 the total number of participants who answered 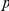 to 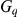. Eq. (4) gives the explicit relationship of the Pearson correlation coefficient of two vectors 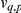 and 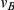 with binary components in terms of the number of respondents 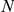, 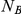, 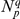 and 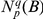. We notice that all the correlation coefficients reported in Table 1 are positive, and some of them reach values larger than 0.3. Furthermore we observe a significant over-representation of respondents in group 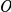 who are either *not sure* (answer 1) about their agreement with statements in section G or just pass over the statements (answer 9). More generally, the total number of over-represented answers in group B is 323 involving 252 questions, while the total number of over-represented answers in group O is 650 involving 254 questions. By taking into account the fact that the total number of questions considered is 258 (including the 6 questions on demographics), and that a given answer cannot be over-represented in both B and O, we conclude that the two groups of respondents show rather distinct patterns. These findings also indicate a greater heterogeneity of respondents outside the Bonferroni network with respect to respondents involved in the network. In Section G of the questionnaire, respondents are also asked to rate their present life according to a quantitative score ranging from the worst possible value (score 0) to the best possible one (score 10) life. It turns out that respondents self rating their present life with a score equal to 8 or higher are over-represented in group 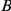. We provide the complete list of questions and answers with a p-value smaller than 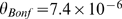 for the groups 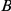 and 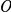, respectively, in data S2. It is also worth to mention here a few other interesting differences between the two groups of respondents. The first difference concerns the sex. Indeed, while the 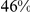 of respondents to the survey are men, this percentage reaches the 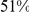 in group 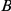. This difference is significant at the Bonferroni level, and indicates that men tend to have a more similar survey profile than women. The second difference concerns the level of education. Indeed we observe that illiterate respondents and respondents with 1–6 years of schooling are both over-represented categories in group 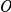. Another factor is the health status. In fact 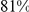 of respondents in group B were never been sick during the 6 months before the survey, with respect to an overall 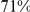 observed for all the respondents. The last factor we comment here is nationality. Netherlands is the only country over-expressed in group B whereas Sweden and Luxembourg are the countries over-expressed in group O. Austrians, Britons and Italians are present in both groups at a level compatible with a null hypothesis of random selection in groups B and O of the size observed.

A more systematic analysis of the factors characterizing groups B and O can be done by looking at the top 5% of the over-represented answers (ranked according to the p-value and then to the correlation in the data S2) in each one of the groups B and O. These are 16 and 33 answers respectively. In the group B, 7 of the 16 top 5% answers deal with the mental status of respondents (sub-section DIV of the questionnaire including 20 questions), and indicate high level of mental status. Other 5 answers in the top 16 are from section G, evaluating satisfaction with life. All these 5 questions present the over-expression of answer 2, which indicates good satisfaction with life. Other 3 questions are from section C of the questionnaire, and indicate good physical health status of respondents in group B. We note that one of these 3 questions is from the subsection “functional activity of daily living”, and indicate that respondents in group B are autonomous in doing some everyday activities people need to do. There is also one over-represented answer from section B of the questionnaire, indicating that respondents in group B almost never feel lonely. The analysis of the 33 over-represented top 5% answers in group O provides an opposite representation of respondents. For this group, 16 out of the top 33 over-represented answers come from section D (13 from DIV). However in this case, these answers indicate low levels of mental status of respondents in this group. There are also 6 answers from section G in the top 33, all consisting of answer 0 to the question, indicative of low levels of satisfaction with life. Six answers come from section C and all indicate that respondents in O present health related issues and problems with functional activity of daily living. Finally, in the top 33 over-represented answers for group O, there is one concerning self-esteem (section DI of the questionnaire), which is low, and two from section F indicating that respondents have problems with normal payments and do not have enough money to buy or obtain small luxuries, or even little “extras”.

The presented results indicate that respondents with a rather homogeneous profile, i.e. respondents in group 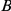, show an average level of life satisfaction that is significantly higher than the one observed in the group 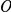 of heterogeneous respondents. In Fig. 1B, we show that the reverse implication is also supported from data, i.e. participants who exhibit high level of life satisfaction are more homogeneous than participants with low level of life satisfaction. Specifically in Fig. 1B, we compare the homogeneity of groups of participants characterized by the same life satisfaction index Z [30] (x-axis). This index can be associated with each participant, and it is obtained by summing the scores assigned to questions 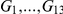 by the participant (407 participants have been excluded from the analysis reported in Fig. 1B because they refused to answer to at least one question of the set 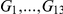). The larger the value of Z, the higher the life satisfaction of the participant (see the text S1 for further details on the index Z and other validated scales). In the y-axis of Fig. 1B, we report the average of the probability mass function 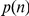 calculated separately for each group of participants by excluding section A of the questionnaire and all questions 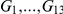. This figure clearly shows that the homogeneity of participants increases with their degree of life satisfaction. We thus provide evidence that Leo Tolstoy's 1877 observation, “Happy families are all alike; every unhappy family is unhappy in its own way” applies to the elderly as well as to families.

The results discussed so far allow us to examine the meaning of *aging successfully*, an ever-discussed topic in geriatrics. In 1997, Rowe and Kahn presented the following definition, widely accepted and used: “successful aging is multidimensional, encompassing the avoidance of disease and disability, the maintenance of high physical and cognitive function, and sustained engagement in social and productive activities” [31]. Other authors have questioned this definition arguing that it overemphasizes the physiological dimension and eludes fundamental components, i.e. connectedness and spirituality. In fact, in the views of the older persons, the multidimensional aspects are broader and include, for example, having autonomy over the place and manner of the final days [32]. Young et al. [33] have recently postulated that positively perceived aging may well coexist with diseases and functional limitations provided that the person is well-supported by solid psychological and/or social mechanisms. The new definition put forward by these authors is: “A state wherein an individual is able to invoke adaptive psychological and social mechanisms to compensate for physiological limitations to achieve a sense of well-being, high self-assessed quality of life, and a sense of personal fulfillment even in the context of illness and disability” [33], [34]. Similarly, Morley suggests that it may be better to allude to “aging successfully”, in reference to people who overcome living with diseases and disability rather than to a “successful aging”, which points to an ideal but infrequent condition of aging without disease [11]. The present analyses tend to support Young's definition [33], because they suggest a major role of mental status with respect to health-related issues. It is worth mentioning that resilience, or the ability to cope with adversity and losses, which can help to reinforce psychological strengths, positive emotions, and regenerative capacities, has been associated with healthy longevity [35]. In the next section, we discuss how, according to the present investigation, resilience is indeed a key aspect to discriminate between different profiles of respondents who, on average, perceive aging positively. Resilience, self-esteem and spheres of control result to be determinant factors in our effort to characterize different clusters of respondents present in the Bonferroni network.

### Clusters of respondents in the Bonferroni network

**Figure 2:**
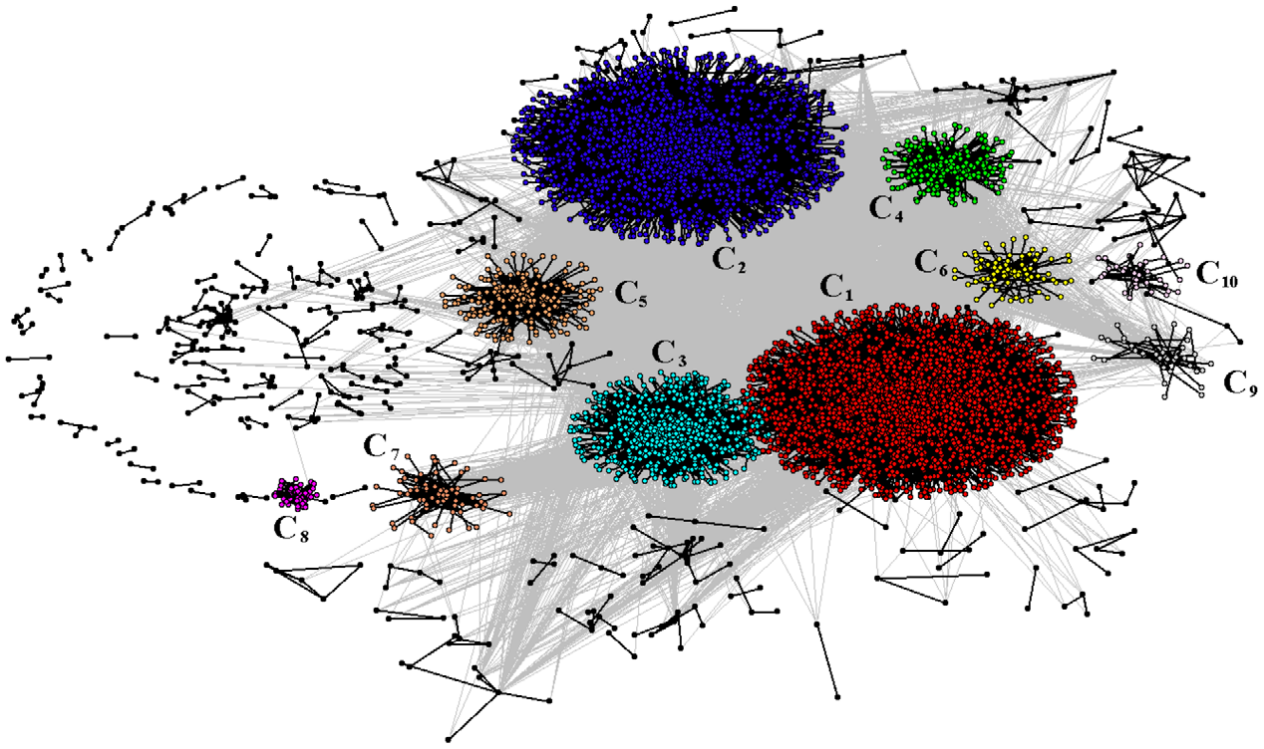
Bonferroni network of respondents to the survey. The colors of vertices allow to distinguish the largest ten clusters of respondents, as partitioned by the Infomap method. The highlighted clusters are labeled as 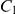,…,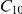 in decreasing order of size. Black links connect respondents belonging to the same cluster, while gray links bridge respondents of different clusters.

**Figure 3:**
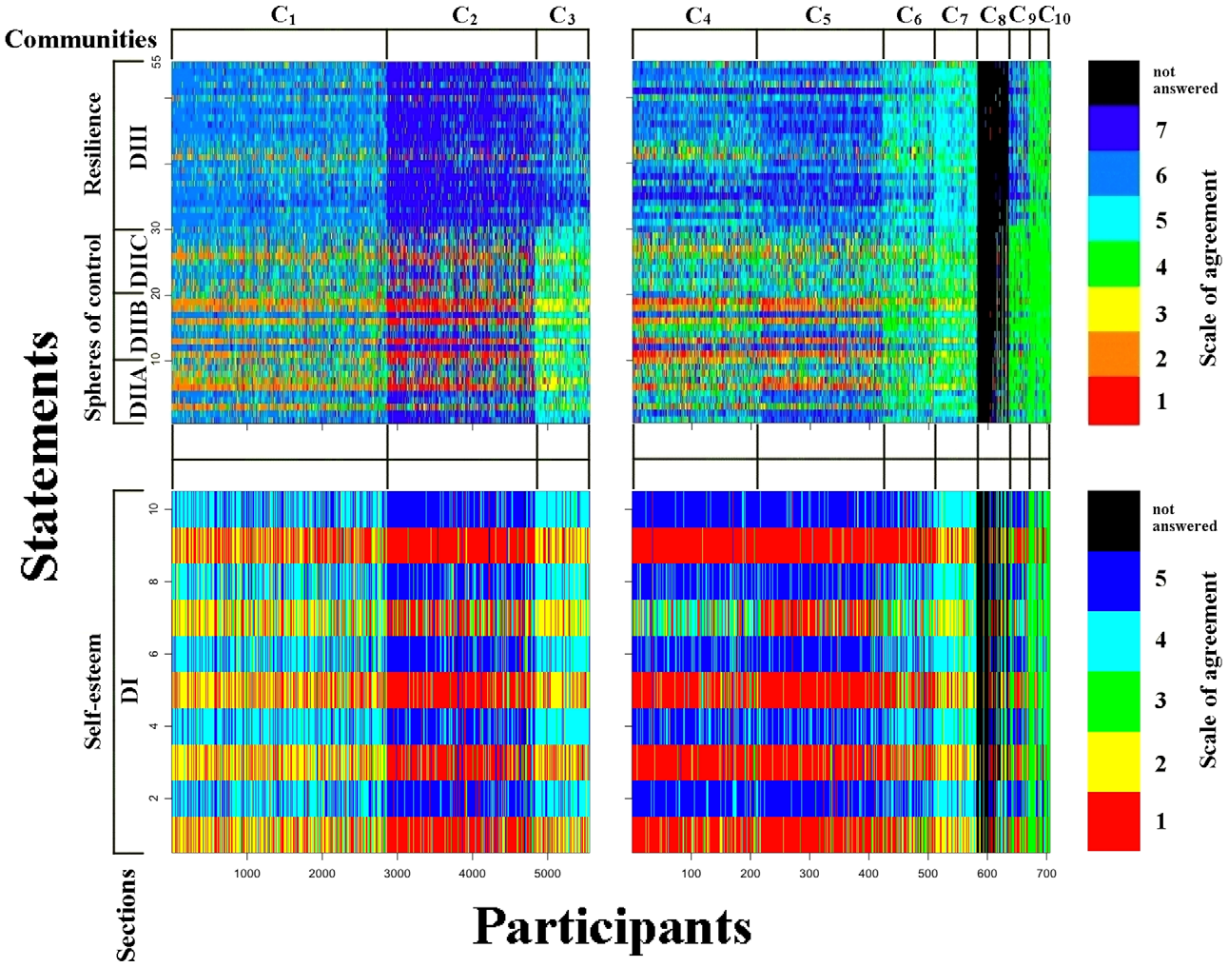
Profile of respondents belonging to the ten largest clusters within the Bonferroni network, as revealed by the Infomap. We only consider the profile of individuals for the subsections DI (self-esteem), DIIA (perceived efficacy), DIIB (interpersonal control), DIIC (sociopolitical control), and DIII (resilience). These subsections are those which better characterize clusters according to the results reported in Table 2. We report the level of agreement of respondents to each statement of the aforementioned subsections. We divide the contour plot in 4 panels. Bottom panels and top panels are separated because the total number of agreement levels – dimension of the Likert scale – to statements in subsection DI is 5, whereas it is 7 for the other subsections. Left panels and right panels separate the largest three clusters of the network (left panels), which include a total of 5,557 respondents, from the remaining 7 clusters, which overall include 705 individuals.

The Bonferroni network has 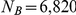 vertices and 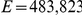 links. Each vertex corresponds to a respondent to the survey and each link indicates a statistically validated similarity between the survey profiles of the two connected individuals. The average vertex degree, which is the average number of connections that each individual forms with the others, is 141.9. The maximum degree is 1,649, which means that this single respondent is connected with 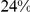 of all the respondents present in the network. In Fig. 2 we show the Bonferroni network. The color of vertices in the figure allows to distinguish the largest 10 clusters of respondents, as partitioned by the Infomap method [36]. This method is considered one of the most effective algorithms for community detection in complex networks [37]. The size of these 10 clusters in decreasing order is: 2,859, 1,973, 725, 217, 205, 86, 73, 54, 36, and 34. Therefore, the total number of respondents belonging to one of these 10 clusters is 6,262, which corresponds to 92% of the number of respondents in the Bonferroni network. In order to characterize these clusters we use an approach analogous to the one already used to characterize the two groups 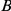 and 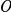 of respondents to the survey. In particular, let us consider a cluster 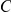 of 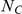 respondents, and indicate the total number of respondents in the Bonferroni network (in cluster 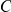) who answer 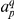 to a given question 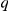 with 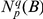 (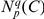). We calculate the p-value for the *over-expression* of answer 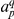 in cluster 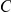 as the probability that at least 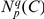 over 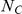 respondents randomly picked in the Bonferroni network give the answer 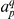 to question 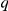. This p-value is again calculated by making use of the hypergeometric distribution: 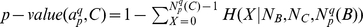. We calculate the p-value for each answer 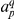 to each question 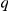 and for each one of the ten largest clusters in the network. The list of over-expressed answers characterizing each cluster is available in the data S2. For each cluster 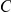 we rank over-expressed answers according to the corresponding p-value in increasing order, and, in case of p-value degeneracy (this is what we always assume for p-values smaller than 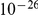), in decreasing order with respect to the correlation coefficient associated with the considered answer and cluster:
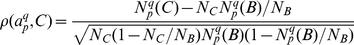
(5)Here Eq. (5) gives the explicit relationship of the Pearson correlation coefficient of two binary vectors 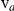 and 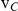 in terms of the number of respondents 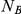, 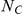, 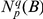 and 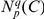. The components of 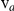 are equal to 1 in correspondence to all the respondents in the Bonferroni network who answered 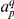 to question 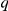, and 0 otherwise, while the components of 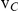 are equal to 1 in correspondence to all the respondents belonging to the cluster 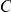, and 0 otherwise. Please notice that the correlation coefficient and the p-value do not carry exactly the same information, i.e. high values of correlation do not always correspond to small p-values. This fact is mainly a consequence of the structure of Eq. (5). In fact if we multiply the quantities 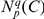, 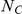, 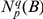 and 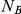 by the same scale factor 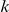 then the correlation 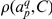 remains the same. This fact implies that we can obtain the same value of the correlation for a sample that is very small and for a sample that is very large. On the contrary, the p-value calculated according to the hypergeometric distribution takes naturally into account the sample size, implying that also a very high value of correlation might not be statistically significant if the sample size is very small. In Table 2, we report information about the top 25% (third column) of the total number (second column) of over-expressed answers in each of the 10 largest clusters. In particular, for each section of the questionnaire, we provide the number of over-expressed answers. In the case of section D, we also detail this information at the level of subsections. The table shows that section D of the questionnaire, which aims to evaluate the mental efficacy of respondents, is the section that most characterizes the revealed clusters of respondents included in the network. In particular relevant subsections are DI (self-esteem), DIIA (perceived efficacy), DIIB (interpersonal control), DIIC (sociopolitical control), and DIII (resilience). In Fig. 3 we show the level of agreement of respondents to all the statements in these subsections. The figure is divided in four panels. The bottom panels report the level of agreement of respondents to statements in subsection DI, while the top panels report their level of agreement to statements in the other subsections. This distinction is necessary because there are five different levels of agreement – Likert scale dimension – to statements in subsection DI, namely *strongly disagree* (Red), *disagree* (Yellow), *undecided* (green), *agree* (Cyan), and *strongly agree* (Blue), while there are seven levels of agreement in the other subsections, ranging from *strongly disagree* (Red) to *strongly agree* (Blue). In the figure, we also use the Black color to indicate those cases in which the respondents refuse to provide any level of agreement. Left panels in the figure show the level of agreement of respondents belonging to the 3 largest clusters in the Bonferroni network, while right panels show results for the remaining 7 clusters. This presentation enhances figure readability because the number of respondents belonging to the 3 largest clusters is about 10 times larger than the total number of respondents in the other 7 clusters. Before moving to the description of the figure and the characterization of clusters, it is important to note that some statements are posed in negative sense. This is the case, for instance, of statements 1, 3, 5, 7, and 9 of section DI (see the questionnaire in data S1). On average, respondents in the largest 10 clusters show at least a fair level of mental efficacy, the only exception being cluster 8, as respondents in this cluster refuse to answer most of the questions. This good level of mental efficacy is in pretty good agreement with the finding that respondents in the Bonferroni network are, on average, significantly more satisfied of their life than people outside the network. However, as we already mentioned, respondents in cluster 8 refuse to indicate their agreement to most of the statements in section D, and in particular to section DIII (resilience), showing a rather non collaborative behavior. For the other clusters, it is interesting to remark comparative differences, in order to clarify their characterizing aspects. Cluster 2 is composed of 1,973 respondents and these respondents show the highest level of mental efficacy. They typically strongly agree (Blue) to positive statements and strongly disagree to negative statements (Red). Cluster 1 is the largest cluster in the network, including 2,859 respondents to the survey. These respondents are positive, but not extremely positive as respondents in cluster 2. Indeed respondents in cluster 1 mostly show a good agreement (level 5 and 6) to positive statements, and mild disagreement (level 2 and 3) or uncertainty (level 4) to negative statements in subsections concerning spheres of control and resilience. A similar profile, although less pronounced, is also observed for statements of subsection DI (self-esteem). Respondents in cluster 3 show a level of agreement to statements concerning the resilience which is intermediate between the profiles of clusters 1 and 2. However, respondents in cluster 3 are rather neutral with respect to spheres of controls, while they show a profile of self-esteem similar to the one observed for cluster 1, i.e. a fair level of self-esteem. Moving to the smaller 7 clusters, we start by discussing the smallest cluster, which is cluster 10 (including just 34 respondents). These respondents are prominently undecided, i.e. their level of agreement to statements of subsection DI is mainly 3, while it is 4 to statements in the other subsections. At difference with respondents in this cluster, the 36 respondents belonging to cluster 9 show high levels of resilience (level 6 and 7 of agreement to statements in subsection DIII). We have already discussed the profile of respondents in cluster 8, who essentially are non collaborative respondents. Cluster 7 is very similar to cluster 3 concerning the agreement of respondents to statements of subsection DI (neutral), but the 73 respondents of cluster 7 are mostly neutral also with respect to the spheres of control, and they show moderately positive value of resilience (level 5 of agreement to statements of subsection DIII). Cluster 6 shows high levels of self-esteem, while it is similar to cluster 7 for what concerns spheres of control and resilience. Cluster 6 is also significantly characterized by answers from sections different than D. First of all 76 out of the 86 respondents in this cluster are from Sweden. They use to talk frequently with friends and/or relatives, and many live in a city. Cluster 5 is composed of 205 respondents. They show a profile of mental efficacy similar to the one of respondents in cluster 2, which is very high, on average. However cluster 5 shows just mild agreement or neutrality to statements of subsection DIIC, i.e. with respect to the sociopolitical control. At last, cluster 4 shows good levels of resilience (mainly level 5, 6, and 7), very good levels of self-esteem (section DI), while it is rather neutral or mild positive with respect to the spheres of controls.

**Table 2:** Characterization of the 10 largest clusters of participants in the Bonferroni network.

Cluster details				All sections of the questionnaire									D section					
Cl.	#	# ov.	Top	A	B	C	D	F	G	ADL	NQ	Country	DI	DIIA	DIIB	DIIC	DIII	DIV
	res.	ex.	25%										(10)	(10)	(10)	(10)	(25)	(20)
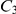	2,859	183	45	0	0	0	44	0	0	0	0	NL	2	9	8	6	19	0
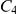	1,973	162	40	0	0	0	40	0	0	0	0	-	3	5	9	0	23	0
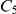	725	160	40	0	0	0	39	0	0	0	0	GB	7	11	10	11	0	0
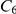	217	95	23	1	1	4	12	4	0	0	1	SE	8	0	0	0	2	2
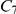	205	52	13	0	0	0	11	0	1	0	0	AT	10	0	0	0	1	0
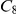	86	80	20	1	1	0	17	0	0	0	0	SE	0	2	2	0	12	1
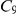	73	76	19	0	0	0	19	0	0	0	0	IT	0	0	1	0	18	0
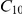	54	126	31	0	0	0	31	0	0	0	0	-	0	1	5	0	25	0
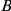	36	39	9	0	0	0	8	0	0	0	0	SE	0	3	2	3	0	0
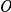	34	80	20	0	0	0	20	0	0	0	0	-	4	0	3	1	12	0

A final comment regards the country over-expression of clusters. It is somehow expected that the national character of each country might show up in a questionnaire like the one we are investigating. In fact, the culture, the heritage, and the traditions, together with more practical issues like the welfare state, might enhance the similarity of profiles associated with respondents from the same country. Indeed respondents belonging to the same cluster also show a high degree of homogeneity with respect to the country they live, as summarized in the table S1. More specifically, Table 2 shows that a single country is in the top 25% over-representations in seven of the ten largest clusters. It is also to mention that one or more countries are also over-expressed in clusters 2, 8 and 10, although none of these over-expressions belongs to the top 25% ones. For example, in cluster 2 three countries are over-expressed, namely Sweden, Luxembourg, and Italy. The degree to which citizens live long in a country is quantified by their life expectancy. According to 1990's statistics, life expectancy was close to 78 years for the countries investigated here. Specifically, life expectancy was ranging from the minimum value of 77.4 years for the Netherlands to the maximal value of 78.2 for Sweden. A comprehensive indicator of quality of life in a country is something several researchers are working on. One of the major contributions in this direction has been the proposal of a Happy Life-Expectancy index [38]. The idea of measuring happy life-expectancy has been made operational by combining information about the life expectancy with survey data on subjective appreciation of life. It is worth noting that according to several reports, e.g. [38], The Netherlands displays a value of happy life-expectancy index higher than the other countries investigated in the present study. In our analysis, The Netherland is the country with more participants present in the Bonferroni network (see Table S1), and this observation is consistent with a high value of happy life-expectancy index, as we have shown that in average respondents involved in the Bonferroni network are more satisfied with life than respondents not included in the network. The role played by countries might also be associated to the questionnaire itself. In fact, differences in the questionnaire language and the fact that different groups of interviewers have collected the data in the different countries might have introduced biases that make more similar respondents from the same country. It must however be recalled that although there were possible differences in the translation of the questionnaire into various languages, this possibility was considered and was controlled by translating the questionnaire not straightforwardly. For example, some questions were calibrated to be culturally/nationally appropriate. The translation and calibration of each language version of the questionnaire was individually tested and then re-calibrated (back) for cross-cultural analysis. As for the interviewers, in order to minimize this issue, all the interviewers received training and specific explanations about each of the sections of the questionnaire in an interactive modality, answering questions and clarifying doubts concerning the compilation of the questionnaire.

### Conclusions

We introduce an unsupervised method to investigate a large survey database. Considering a survey as a bipartite network in which respondents are linked to the actual answers they provide allows one to investigate and statistically assess the degree of similarity among answers profiles of respondents. This approach naturally highlights the presence of clusters of respondents independently of demographic factors. Our analysis of the survey on “Aging Well” of the ESAW project shows that respondents can be partitioned in two large groups by statistically validating the co-occurrence of the same pattern of answers among respondents. The first group is formed by respondents with a positive perception of aging, while the second set includes respondents much less satisfied with their life conditions. The profiles of respondents of the first group, who present a quite positive self-perception of aging, are significantly more homogeneous than the profiles of respondents of the second group. Key indicators of an optimistic perception of aging are a good health and mental status; however a non negligible role of sex, education level and, in some cases, nationality, should also be considered. The first group of respondents are also partitioned in an unsupervised way according to the similarity profile of survey answers. The clusters of respondents thus obtained are prominently characterized by different levels of resilience, self-esteem and perception of the spheres of control.

A negative self-perception of aging may put people at risk or may be hallmark of undetected depression. Even if depression is frequently observed in old age [39], [40], it is not age-related and there is no reason to believe that it may be part of normal aging. Depression is associated with physical illness and disability, life events, loneliness, social isolation, and it is projected to be one of the leading causes of disease burden in older populations by the year 2030 [41]. Therefore, it is crucial to untangle the determinants of a negative perception of aging, likewise the factors associated with a positive perception, in order to reduce morbidity, demand on health and social services and the cost of community care. Our results showing that resilience, self-esteem and perception of the spheres of control characterize the clusters of respondents with a positive perception of aging suggest that these parameters may help to identify subjects at risk of depression. Since a positive perception of life can have major implications on diverse dimensions of societal development (i.e., happy nations are more likely to be successful, not only from an economic viewpoint, than unhappy nations) [42], these specific cluster characteristics will be further explored in future studies.

## Supporting Information

Text S1 **Description of the database filtering and modifications.** We provide the details of all modifications done to the original version of the questionnaire. We have modified the original database in order to remove possible sources of statistical bias influencing the analyses. The modification process consisted of grouping some answers together in order to allow a description of answers to any given question in terms of categorical variables. We also summarize the validated scales used in the questionnaire together with the section in which they appear and the reference to the paper in which the scale has been originally proposed.
(DOC)

Data S1 **English version of the questionnaire used to administer the survey.** It includes the English version of the questionnaire, together with the list of activities considered in Section E. The questionnaire has been submitted to each participant in his own language. In order to minimize the biases introduced by possible differences in the translation of the questionnaire, some questions were calibrated to be culturally/nationally appropriate. The translation and calibration of each language version of the questionnaire was individually tested and then re-calibrated (back) for cross-cultural analysis. As for the interviewers, in order to minimize this issue, all the interviewers received training and specific explanations about each of the sections of the questionnaire in an interactive modality, answering questions and clarifying doubts concerning the compilation of the questionnaire.
(DOC)

Data S2
**Characterization of different groups of participants according to the over-representation and under-representation of answers in each group.** It includes the complete list of answers with a p-value smaller than the bonferroni threshold characterizing the groups 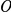 (Worksheet Group B characterization) and 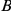 (Worksheet Group 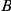 characterization). In the third worksheet (Worksheet Characterization clusters 1–10) we give all details of the attributes that characterize the largest 10 clusters of the group 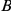 (see also Fig. 2).(XLS)

Table S1
**Summary of the characterization of the 10 largest clusters of participants in the Bonferroni network.** It describes the relative relevance of different sections of the questionnaire in characterizing the 10 largest clusters within group 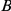 of participants. It also provides details about the fact that respondents belonging to the same cluster show a high degree of homogeneity with respect to the country they live.(XLS)
