# Supplementary material for: Happy Aged People Are All Alike, While Every Unhappy Aged Person Is Unhappy in Its Own Way
Source: PLoS One. 2011 Sep 8;6(9):e23377. doi: 10.1371/journal.pone.0023377 (PMC3169534; doi:10.1371/journal.pone.0023377)
Supplement: Text S1 — Description of the database filtering and modifications. We provide the details of all modifications done to the original version of the questionnaire. We have modified the original database in order to remove possible sources of statistical bias influencing the analyses. The modification process consisted of grouping some answers together in order to allow a description of answers to any given question in terms of categorical variables. We also summarize the validated scales used in the questionnaire together with the section in which they appear and the reference to the paper in which the scale has been originally proposed. (DOC) [file pone.0023377.s001.doc]

Supplementary Information to the manuscript:

**Happy aged people are all alike, while every unhappy aged person is unhappy in its own way**

In the paper we have analyzed the results of a survey of N=12,478 people aged 50 to 90, from 6 West European countries, in the context of the collaborative cross-cultural “European study of adult well being” (ESAW). The project aimed to develop a globally applicable model of “Aging Well”', estimating the direct causal contribution of five key components, personal characteristics and culture, to the outcome variable “Aging Well”:

1. physical health and functional status,
2. self-resources,
3. material security,
4. social support resources, and
5. life activity.

Six West European countries participated in the study:

- - Austria (AT) with 2,111 respondents.
  - Italy (IT) with 2,018 respondents.
  - Luxembourg (LU) with 2,145 respondents.
  - The Netherlands (NL) with 1,934 respondents.
  - Sweden (SE) with 2,417 respondents.
  - United Kingdom (GB) with 1,853 respondents.

Thus, the six countries range from Scandinavia to the Mediterranean. In its original form, the database comprehends all the answers given by respondents to a 504-item questionnaire divided in 7 sections. **Section A** provides background information about the respondent, e.g. home country, age, education, living in a city or in countryside. **Section B** deals with social support resources. **Section C** provides information about physical health and functional status. **Section D** aims at evaluating the mental efficacy of the respondents. **Section E** considers life activity, while **section F** considers material security. Finally, **Section G** evaluates the self-perception of aging.

The English version of the questionnaire is provided in the supplementary information file Questionnaire.doc, together with the list of activities considered in Section E of the questionnaire.

**1. DATABASE MODIFICATIONS**

We have modified the original database in order to remove possible sources of statistical bias influencing the analyses. The modification process consisted of grouping some answers together in order to allow a description of answers to any given question in terms of categorical variables. The modification process has been carried out in a series of steps that we illustrate in the following sections.

## 1.1 Aggregation of answers

1. **Question A4_b** – the possible answers to this question have been grouped as follows:
   1. From 51 year old to 60 years old – answer 1
   2. From 61 year old to 70 years old – answer 2
   3. From 71 year old to 80 years old – answer 3
   4. From 81 year old to 90 years old – answer 4
2. **Question C1** – the possible answers to this question have been grouped as follows:
   1. 0 days – answer 0
   2. 1 day – answer 1
   3. From 2 days to 6 days– answer 2
   4. From 7 days to 12 days– answer 3
   5. From 13 days to 24 days– answer 4
   6. More than 25 days– answer 5
   7. No answer– answer 999
3. **Question C3** – see question C1
4. **Question C4** – see question C1
5. **Question C7** - the possible answers to this question have been grouped as follows:
   1. 0 drug – answer 0
   2. 1 drug – answer 1
   3. 2 drugs– answer 2
   4. More than 3 drugs – answer 3
   5. No answer– answer 99
6. **Question C16_b** – the possible answers to this question have been grouped as follows:
   1. 0 cigarettes – answer 0
   2. 10 cigarettes – answer 1
   3. 20 cigarettes – answer 2
   4. 30 cigarettes – answer 3
   5. 40 cigarettes – answer 4
   6. 40 cigarettes – answer 5
   7. 50 cigarettes – answer 6
   8. 60 cigarettes – answer 7
   9. More than 60 cigarettes – answer 8
   10. No answer – answer 99
7. No answer– answer 99
8. **Question C17_a1** - the possible answers to this question have been grouped as follows:
   1. From 0 to 4 hours – answer 1
   2. 5 hours – answer 2
   3. 6 hours – answer 3
   4. 7 hours – answer 4
   5. 8 hours – answer 5
   6. 9 hours – answer 6
   7. More than 10 hours – answer 7
   8. No answer – answer 99
9. **Question C17_b1** – the possible answers to this question have been grouped as follows:
   1. From 12:00 to 19:00 – answer 1
   2. From 20:00 to 22:00 – answer 2
   3. From 23:00 to 24:00 – answer 3
   4. From 01:00 to 02:00 – answer 4
   5. After 02:00 – answer 5
10. **Question C17_c1** – the possible answers to this question have been grouped as follows:
    1. Before 04:00 – answer 1
    2. From 4:00 to 6:00 – answer 2
    3. From 6:00 to 8:00 – answer 3
    4. From 8:00 to 10:00 – answer 4
    5. After 10:00 – answer 5
11. **Question F13_c1 and F13_c2 -** the possible answers to these questions have been aggregated as follows:
    1. 0 km – answer 0
    2. from 1 to 2 km – answer 1
    3. from 3 to 5 km – answer 2
    4. from 6 to 10 km – answer 3
    5. from 11 to 20 km – answer 4
    6. from 21 to 30 km – answer 5
    7. more than 30 km – answer 6

## 1.2 Elimination and/or aggregation of questions

1. **Question B9** – it has been eliminated because it was redundant with respect to questions **B9_a** and **B9_b.**
2. **Question C12_13** – it has been dropped, because the answer was not quantitative.
3. **Question C14_b** – it has been dropped, because the answer was not quantitative.
4. **Question C15_a** – it has been eliminated because it was redundant with respect to question **C15_b.**
5. **Question C15_b1 and C15_b2** – These two questions have been aggregated as follows:

each answer to question C15_b2 has been multiplied by the answer to question C15_b1. Then the results have been aggregated as follows:

- 1. 0 – answer 1
  2. Less than 1 – answer 2
  3. Less than 2 – answer 3
  4. Less than 4 – answer 4
  5. More than 4 – answer 5

1. **Question C15_c1 and C15_c2** – These two questions have been aggregated as follows:

each answer to question C15_c2 has been multiplied by the answer to question C15_c1. Then the results have been aggregated as follows:

- 1. 0 – answer 1
  2. Less than 1 – answer 2
  3. Less than 2 – answer 3
  4. Less than 4 – answer 4
  5. More than 4 – answer 5

1. **Question C15_d1 and C15_d2** – These two questions have been aggregated as follows:

each answer to question C15_d2 has been multiplied by the answer to question C15_d1. Then the results have been aggregated as follows:

- 1. 0 – answer 1
  2. Less than 1 – answer 2
  3. Less than 2 – answer 3
  4. Less than 4 – answer 4
  5. More than 4 – answer 5

1. **Question C16_a** – it has been dropped because it was redundant with respect to question **C16_b.**
2. **Question C17_a2 –** it has been dropped. Only the information relative to question C17_a1 has been considered.
3. **Question C17_b2 –** it has been dropped. Only the information relative to question C17_a1 has been considered.
4. **Question C17_c2 –** it has been dropped. Only the information relative to question C17_a1 has been considered.
5. **Questions F2 and F2_b** – These two questions have been aggregated as follows:
   1. No answer – answer 0
   2. never employed – answer 1
   3. Housewife/Househusband – answer 2
   4. Professional – answer 3
   5. Manager or proprietor– answer 4
   6. Farmer– answer 5
   7. Clerical, sales or technical– answer 6
   8. Skilled, foreman– answer 7
   9. Semi-skilled, operative– answer 8
   10. Service worker– answer 9
   11. Unskilled worker– answer 10
   12. Farm laborer– answer 11
6. **Questions F3 and F3_b** – These two questions have been aggregated as follows:
   1. No answer – answer 0
   2. never employed – answer 1
   3. Housewife/Househusband – answer 2
   4. Professional – answer 3
   5. Manager or proprietor– answer 4
   6. Farmer– answer 5
   7. Clerical, sales or technical– answer 6
   8. Skilled, foreman– answer 7
   9. Semi-skilled, operative– answer 8
   10. Service worker– answer 9
   11. Unskilled worker– answer 10
   12. Farm laborer– answer 11
7. **Questions F4** – It has been dropped because the money unit was not given
8. **Questions F7, F7_a, F7_b** – These questions have been aggregated as follows:
   1. F7=9 F7_a=9 F7_b=9 answer 9
   2. F7=1 F7_a=1 F7_b=9 answer 1
   3. F7=1 F7_a=2 F7_b=9 answer 2
   4. F7=1 F7_a=9 F7_b=9 answer 3
   5. F7=0 F7_a=9 F7_b=1 answer 4
   6. F7=0 F7_a=9 F7_b=2 answer 5
   7. F7=0 F7_a=9 F7_b=3 answer 6
   8. F7=0 F7_a=9 F7_b=9 answer 7
9. **Question F13_c1 and F13_c2 -** These questions have been aggregated as follows:
10. 0 km– answer 1
11. From 1 to 2 km – answer 2
12. From 3 to 5 km– answer 3
13. From 6 to 10 km – answer 4
14. From 11 to 20 km – answer 5
15. From 20.92 to 30 km – answer 6
16. More than 30 km – answer 7

Moreover, we have added two new questions with respect to the original questionnaire: **Question** **C6_SUM** and **Question** **C8_SUM**. These questions summarize the information of **Question C6** and **Question C8** by introducing the total number of drugs (C6_SUM) and diseases (C8_SUM.). Essentially C6_SUM and C8_SUM are the sum of all answers of type ***yes*** to question C6 and C8, respectively.

As a result, after these modifications the database comprehends 490 questions divided in 7 sections for a total number of 2663 possible answers.

## 1.3 Poorly answered questions

To avoid that spurious similarity among individuals arise due to not answered questions, we have removed from the database all the questions such that at least 10% of respondents refused to answer.

Below we report the list of questions that have been removed from the database with the indication of the number of people who refused to answer (null answer) the question. For the sake of completeness we report the label associated with each null answer, e.g. 9, 99, and the percentage of people giving that answer.

1. **Question ADL15B**; *null answer*: 9; *number of null answers*: 11299; *percentage*: 91%
2. **Question EI4**; *null answer*: 9; *number of null answers*: 10117; *percentage*: 81%
3. **Question ADL15A**; *null answer*: 9; *number of null answers*: 9221; *percentage*: 74%
4. **Question F6-13**; *null answer*: 9; *number of null answers*: 8860; *percentage*: 71%
5. **Question F6-11**; *null answer*: 9; *number of null answers*: 8495; *percentage*: 68%
6. **Question F6-4**; *null answer*: 9; *number of null answers*: 4797; *percentage*: 38%
7. **Question F6-9**; *null answer*: 9; *number of null answers*: 4538; *percentage*: 36%
8. **Question C15E**; *null answer*: 9; *number of null answers*: 3285; *percentage*: 26%
9. **Question A4-1**; *null answer*: 99; *number of null answers*: 3015; *percentage*: 24%
10. **Question F13A-5**; *null answer*: 9; *number of null answers*: 2802; *percentage*: 22%
11. **Question F13B**; *null answer*: 9; *number of null answers*: 2757; *percentage*: 22%
12. **Question EI3**; *null answer*: 9; *number of null answers* 2682; *percentage*: 21%
13. **Question EII5-18**; *null answer*: 9; *number of null answers*: 2591; *percentage*: 21%
14. **Question EII5-16**; *null answer*: 9; *number of null answers*: 2591; *percentage*: 21%
15. **Question EII5-19**; *null answer*: 9; *number of null answers*: 2585; *percentage*: 21%
16. **Question EII5-15**; *null answer*: 9; *number of null answers*: 2585; *percentage*: 21%
17. **Question EII5-14**; *null answer*: 9; *number of null answers*: 2584; *percentage*: 21%
18. **Question EII5-13**; *null answer*: 9; *number of null answers*: 2582; *percentage*: 21%
19. **Question EII5-12**; *null answer*: 9; *number of null answers*: 2580; *percentage*: 21%
20. **Question EII5-17**; *null answer*: 9; *number of null answers*: 2579; *percentage*: 21%
21. **Question EII5-10**; *null answer*: 9; *number of null answers*: 2579; *percentage*: 21%
22. **Question EII5-6**; null answer: 9; *number of null answers*: 2578; *percentage*: 21%
23. **Question EII5-11**; null answer: 9; *number of null answers*: 2577; *percentage*: 21%
24. **Question EII5-**8; null answer: 9; *number of null answers*: 2573; *percentage*: 21%
25. **Question EII5-7**; null answer: 9; *number of null answers*: 2573; *percentage*: 21%
26. **Question EII5-9**; null answer: 9; *number of null answers*: 2572; *percentage*: 21%
27. **Question EII5-2**; null answer: 9; *number of null answers*: 2572; *percentage*: 21%
28. **Question EII5-4**; null answer: 9; *number of null answers*: 2565; *percentage*: 21%
29. **Question EII5-5**; null answer: 9; *number of null answers*: 2564; *percentage*: 21%
30. **Question EII5-3**; null answer: 9; *number of null answers*: 2559; *percentage*: 21%
31. **Question F13A-3**; null answer: 9; *number of null answers*: 2557; *percentage*: 20%
32. **Question EII5-1**; null answer: 9; *number of null answers*: 2550; *percentage*: 20%
33. **Question B2-10**; null answer: 9; *number of null answers*: 2517; *percentage*: 20%
34. **Question F6-12**; null answer: 9; *number of null answers*: 2476; *percentage*: 20%
35. **Question F6-8**; null answer: 9; *number of null answers*: 2471; *percentage*: 20%
36. **Question F6-5**; null answer: 9; *number of null answers*: 2464; *percentage*: 20%
37. **Question F6-3**; null answer: 9; *number of null answers*: 2419; *percentage*: 19%
38. **Question F6-14**; null answer: 9; *number of null answers*: 2309; *percentage*: 19%
39. **Question C18B**; null answer: 9; *number of null answers*: 2164; *percentage*: 17%
40. **Question EIIIB-1A**; null answer: 99; *number of null answers*: 1982; *percentage*: 16%
41. **Question EIIIB-3A**; null answer: 99; *number of null answers*: 1954; *percentage*: 16%
42. **Question EIIIB-2A**; null answer: 99; *number of null answers*: 1919; *percentage*: 15%
43. **Question B9B**; null answer: 9; *number of null answers*: 1783; *percentage*: 14%
44. **Question B9A**; null answer: 9; *number of null answers*: 1677; *percentage*: 13%
45. **Question F17**; null answer: 9; *number of null answers*: 1603; *percentage*: 13%
46. **Question C15D-1**; null answer: 999; *number of null answers*: 1583; *percentage*: 13%
47. **Question EII4**; null answer: 9; *number of null answers*: 1480; *percentage*: 12%
48. **Question EIIC-A all**; null answer: 99; *number of null answers*: 1286; *percentage*: 10%
49. **Question EIIC-B all**; null answer: 99; *number of null answers*: 1286; *percentage*: 10%

As a result, after this second run of modifications the database comprehends 426 questions divided in 7 sections.

## 1.3 Removal of Section E

Section E was finally removed because of the different structure of questions in it. In fact, questions in this section describe *life activities* that are specific to each respondent. Each respondent was free to indicate one or more activities for each posed question. No limit was given to the number of activities. Moreover, the order by which the activities had to be indicated was not predetermined. Having that, we couldn’t find a sound way to reshape the set of answers given by respondents to questions in section E, in order to allow a description in terms of categorical variables without introducing spurious correlations among answers.

After removing section E, we were left with 258 questions, all of them having answers in a form of categorical variables.

**2. VALIDATED SCALES**

Many questions proposed in the questionnaire are taken from batteries of tests, which are well known in the literature. Below, we summarize the validated scales present in the questionnaire together with the section in which they appear and the reference to the paper in which the scale has been proposed.

**SECTION B** (from question NQ1 to NQ8) - Practitioner Assessment of Network Type (PANT)

Wenger, G. C. and I. Tucker (2002). “*Using network variation in practice: identification of support network type*” Health Soc Care Community,**10** (1): 28.

**SECTION C** (from question ADL1 to ADL15b) - ADL/IADL

Katz, S., A. B. Ford, et al. (1963). "*Studies of Illness in the Aged. The Index of Adl: A Standardized Measure of Biological and Psychosocial Function*" Jama, **185**: 914.

Lawton E.P. and Brody E.M. (1969). “*Assessment of older people - self-maintaining and instrumental activities of daily living”.*  Gerontologist, **9** (3p1): 179.

**SECTION C** (from question ADL1 to ADL15b) - Multidimensional Functional Assessment Questionnaire (OMFAQ)

Fillenbaum, G. G. and M. A. Smyer (1981). "*The development, validity, and reliability of the OARS multidimensional functional assessment questionnaire*." J Gerontol **36**(4): 428.

**SECTION D I** - Rosenberg Self-Esteem Scale

Rosenberg, M. (1965). Society and the adolescent self-image. Princeton, NJ: Princeton University Press.

**SECTION D II** - Spheres of Control Battery

Paulhus, D. L., P. D. Harms, et al. (2003). “*The over-claiming technique: measuring self-enhancement independent of ability*.” J Pers Soc Psychol **84**(4): 890.

**SECTION D III** - Resilience scale

Wagnild, G. M. and H. M. Young (1993). “*Development and psychometric evaluation of the Resilience Scale*.” J Nurs Meas **1**(2): 165.

**SECTION G** (from question G1 to G13) - Life Satisfaction Index Z

Neugarten, B. L., R. J. Havighurst, et al. (1961). “*The measurement of life satisfaction*.” J Gerontol **16**: 134.

**SECTION G** (question G14) - Cantril’s Self Anchoring Scale

Cantril, H. (1965). The pattern of human concerns. New Brunswick, NJ: Rutgers University Press.
