# Supplementary material for: Happy Aged People Are All Alike, While Every Unhappy Aged Person Is Unhappy in Its Own Way
Source: PLoS One. 2011 Sep 8;6(9):e23377. doi: 10.1371/journal.pone.0023377 (PMC3169534; doi:10.1371/journal.pone.0023377)
Supplement: Data S1 — English version of the questionnaire used to administer the survey. It includes the English version of the questionnaire, together with the list of activities considered in Section E. The questionnaire has been submitted to each participant in his own language. In order to minimize the biases introduced by possible differences in the translation of the questionnaire, some questions were calibrated to be culturally/nationally appropriate. The translation and calibration of each language version of the questionnaire was individually tested and then re-calibrated (back) for cross-cultural analysis. As for the interviewers, in order to minimize this issue, all the interviewers received training and specific explanations about each of the sections of the questionnaire in an interactive modality, answering questions and clarifying doubts concerning the compilation of the questionnaire. (DOC) [file pone.0023377.s002.doc]

**AGING WELL - A STUDY OF ADULT WELL -BEING**

# SECTION A: BACKGROUND INFORMATION

[Note to Interviewers and Interviewees: The United Nations Education, Scientific and Cultural Organization (UNESCO) uses the terms pre-primary for kindergarten, primary/basic education for elementary, secondary for middle school, and tertiary for senior/ high school. In question 4d, if the date of birth of parents is going to present a problem, you will have to decide what you want to do with that question for your own country].

**Country Code:** ________________________

**Interviewer Code**: ______

**Subject** #:_____________________________

**Date of Test:**  / / 20

Month / Day / Year

**A1**. Subject's Location

                                City AND                    State, Province, or Region (as applies)

**A2**. Sex

_____ (1) Male

_____ (2) Female

**A3**. Race / ethnicity/ or religion: (As an example, in the USA the categories are as follows):

_____(1) White (Caucasian)
_____(2) Black/African American

_____(3) Asian American

_____(9) Other – Specify

**A4**. Birth Date and Age. When were you born?

A4_1 ___________ Month

A4_2 ___________ Day

A4_3 ___________ Year

(BIRTH DATE BASED ON GRECO-ROMAN CALENDAR YEAR.)

[A code will be set for those who do not know their date of birth]

**A4B** How old are you now? _____________________________________

**A5**. How far did you go in school?

_____(1) Primary/ basic/ elementary education (1-6 years of schooling)

_____(2) Secondary/ middle/ intermediate education (7-9 years of schooling)

_____(3) Tertiary/ baccalaureate/ high school (10-12/13 years of schooling)

_____(4) Post high school business or trade school

_____(5) College (1-4 years)

_____(6) Graduate education (5-7 years)

_____(7) Post Graduate (7+)

_____(8) No schooling/ illiterate

_____(9) Not answered

**AGING WELL - A STUDY OF ADULT WELL-BEING**

**SECTION B**: SOCIAL SUPPORT RESOURCES

This part of the questionnaire asks you some questions about your family and friends.

**B1.** Are you single, married, widowed, divorced or separated-- RIGHT NOW?

_____(1) Single
_____(2) Married
_____(3) Widowed
_____(4) Divorced
_____(5) Separated
_____(9) Not answered

**B2.** Who lives with you?  [CHECK "YES" OR "NO" FOR EACH OF THE FOLLOWING.]

| **YES**  **(1)** | **NO**  **(0)** | **Not Answered**  **(9)** |  |
| --- | --- | --- | --- |
|  |  |  | B2_1 No one |
|  |  |  | **B2_2** Husband/wife/significant other (male or female with whom you share a committed and intimate relationship) |
|  |  |  | **B2_3** Children |
|  |  |  | **B2_4** Grandchildren |
|  |  |  | **B2_5** Parents |
|  |  |  | **B2_6** Grandparents |
|  |  |  | **B2_7** Brothers and sisters |
|  |  |  | **B2_8** Other relatives (does not include in-laws covered in the above categories.) |
|  |  |  | **B2_9** Friends |
|  |  |  | **B2_10** Non-related paid* helpers (*includes free room) |
|  |  |  | **B2_11** Other, specify:__________________________________ |

**B3**. How many people do you know well enough to visit in their homes?

___(3) Five or more
___(2) Three to four
___(1) One to two
___(0) None at all
___(9) Not answered

**B4**. About how many times did you talk to someone--friends, relatives, or others in the past week (either in person, on the telephone, or e-mail)

___(3) Once a day or more
___(2) 2-6 times
___(1) Once
___(0) Not at all
___(9) Not answered

**B5.** How many times during the past week did you spend some time with someone who does not live with you, that is you went to see them or they came to visit you, or you went out to do things together?

___(3) Once a day or more
___(2) 2-6 times
___(1) Once
___(0) Not at all
___(9) Not answered

**B6**. Do you have someone you can trust and confide in?

___(1) Yes
___(0) No
___(9) Not answered

**B7**. Do you find yourself feeling lonely quite often, sometimes, or almost never?

___(0) Quite often
___(1) Sometimes
___(2) Almost never
___(9) Not answered

**B8.** Do you see your relatives and friends as often as you want ?

___(1) Yes

___(0) No

___(9) Not answered

**B9.** Is there someone who would take care of you if you were sick or disabled?

___(1) Yes

___(0) No one willing and/or able to help
___(9) Not answered

IF "YES" ANSWER B9a. AND B9b. below

**B9a.** Is there:

___(1) Someone who would take care of you for as long as you needed

___(2) Someone who would take care of you for a short time (a few weeks to six months)

___(3) Someone who would take care of you now and then (such as, take you to the doctor or fix your lunch, etc.)

___(9) Not answered or, No one to take care of me

**B9b**. Who is this person?

___(1) Husband/wife/significant other

___(2) Brother/Sister

___(3) Children

___(4) Grandchildren

___(5) Other relatives

___(6) Friends

___(7) Other

___(9) Not Answered

**PRACTITIONER ASSESSMENT OF NETWORK TYPE (PANT)**

Instructions: Now I am going to ask you some more questions about your social relationships.
[Ask all questions, give response alternatives, and check the appropriate response.]

**NQ1.** How far away, in distance, does your nearest child or other relative live? (Do not include spouse.)

_____ (0) No relatives
_____ (1) Same house/within 1 mile
_____ (2) 1-5 miles
_____ (3) 6-15 miles
_____ (4) 16-50 miles
_____ (5) 50+ miles

_____(9) Not answered

**NQ2.** If you have any children, where does your nearest child live?

_____ (0) No children
_____ (1) Same house/within 1 mile
_____ (2) 1-5 miles
_____ (3) 6-15 miles
_____ (4) 16-50 miles
_____ (5) 50+ miles

_____ (9) Not answered

**NQ3.** If you have any living sisters or brothers, where does your nearest sister or brother live?

_____ (0) No sisters or brothers
_____ (1) Same house/within 1 mile
_____ (2) 1-5 miles
_____ (3) 6-15 miles
_____ (4) 16-50 miles
_____ (5) 50+ miles

_____ (9) Not answered

**NQ4.** How often do you see any of your children or other relatives to speak to?

_____ (0) Never/no relative
_____ (1) Daily
_____ (2) 2-3 times a week
_____ (3) At least weekly
_____ (4) At least monthly
_____ (5) Less often

_____ (9) Not answered

**NQ5.** If you have friends in this community/ neighbourhood, how often do you have a chat or do something with one of your friends?

_____ (0) Never/no friends
_____ (1) Daily
_____ (2) 2-3 times a week
_____ (3) At least weekly
_____ (4) At least monthly
_____ (5) Less often

_____ (9) Not answered

**NQ6.** How often do you see any of your neighbours to have a chat with or do something with?

_____ (0) No contact with neighbours
_____ (1) Daily
_____ (2) 2-3 times a week
_____ (3) At least weekly
_____ (4) At least monthly
_____ (5) Less often

_____ (9) Not answered

**NQ7.** Do you attend any religious meetings?

_____ (0) Yes, regularly
_____ (1) Yes, occasionally
_____ (2) No

_____ (9) Not answered

**NQ8.** Do you attend meetings of any community/neighbourhood or social groups, such as old people's clubs, lectures or anything like that?

_____ (0) Yes, regularly
_____ (1) Yes, occasionally
_____ (2) No

_____ (9) Not answered

[copyright, G. Clare Wenger]

**GENERAL SATISFACTION WITH RELATIONSHIPS**

**BI.** How satisfied are you with your family relationships?

[READ THE RESPONSES AND PLACE A CHECK IN THE BOX THAT MATCHES THE SUBJECT'S RESPONSE.]

| **Highly Dissatisfied**  **BI_1** | **Moderately Dissatisfied**  **BI_2** | **Undecided or Neither**  **BI_3** | **Moderately Satisfied**  **BI_4** | **Highly Satisfied**  **BI_5** | **Not Answered**  **BI_9** |
| --- | --- | --- | --- | --- | --- |

**BII.** How satisfied are you with your friendship relationships?

[READ THE RESPONSES AND PLACE A CHECK IN THE BOX THAT MATCHES THE SUBJECT'S RESPONSE.]

| **Highly Dissatisfied**  **BII_1** | **Moderately Dissatisfied**  **BII_2** | **Undecided or Neither**  **BII_3** | **Moderately Satisfied**  **BII_4** | **Highly Satisfied**  **BII_5** | **Not Answered**  **BII_9** |
| --- | --- | --- | --- | --- | --- |

**AGING WELL - A STUDY OF ADULT WELL-BEING**

SECTION C: PHYSICAL HEALTH & FUNCTIONAL STATUS

This part of the questionnaire asks you some questions about your health and daily living skills. The first part is about your health. Please fill in the requested information, or place a check or circle for the correct response.

**C1.** About how many times have you seen a doctor [or health professional] during the past six months other than as an inpatient in a hospital? ________ Put number of times (Not Answered=999)

**C2.** During the past six months, how many days were you so sick that you were unable to carry on your usual activities--such as going to work or working around the house?

___(0) None

___(1) A week or less

___(2) More than a week but less than one month

___(3) 1-3 months

___(4) 4-6 months

___(9) Not answered

**C3.** How many days in the past six months were you in a hospital for physical or emotional health problems?

________ Days (Not Answered=999)

**C4.** How many days in the past six months were you in a nursing home, or rehabilitation center for physical health problems?

________ Days (Not Answered=999)

**C5**. Do you feel that you need medical care or treatment beyond what you are receiving at this time?

___(1) Yes

___(0) No

___(9) Not answered

**C6.** I have a list of medicines (prescription drugs, over the counter drugs, or herbal preparations) that some people take. Would you please tell me if you've taken any of the following in the past month.

| **NOT**  **ANSWERED (9)** | **YES**  **(1)** | **NO**  **(0)** | **[CHECK "YES" OR "NO" FOR EACH MEDICINE.]** |
| --- | --- | --- | --- |
|  |  |  | C6_1 Arthritis medication |
|  |  |  | **C6_2** Prescription pain killer (other than above) |
|  |  |  | **C6_3** High blood pressure medicine |
|  |  |  | **C6_4** Pills to make you lose water or salt (water pills) |
|  |  |  | **C6_5** Digitalis pills for the heart |
|  |  |  | **C6_6** Nitroglycerin tablets for chest pain |
|  |  |  | **C6_7** Blood thinner medicine (anticoagulants) |
|  |  |  | **C6_8** Drugs to improve circulation |
|  |  |  | **C6_9** Insulin injections for diabetes |
|  |  |  | **C6_10** Pills for diabetes |
|  |  |  | **C6_11** Prescription ulcer medicine |
|  |  |  | **C6_12** Seizure medications (like Dilantin) |
|  |  |  | **C6_13** Thyroid pills |
|  |  |  | **C6_14** Cortisone pills or injections |
|  |  |  | **C6_15** Antibiotics |
|  |  |  | **C6_16** Tranquilizers or nerve medicine |
|  |  |  | **C6_17** Prescription sleeping pills (once a week or more) |
|  |  |  | **C6_18** Hormones, male or female (for example birth control pills, hormone replacement therapy) |
|  |  |  | **C6_19** Medications for incontinence |
|  |  |  | **C6_20** Medication for constipation |

**C7.** How many other prescription drugs, over the counter drugs, or herbal preparations have you taken in the past month? (00=if none; 99=Not Answered)

**C8.** Do you have any of the following health conditions at the present time?

[CHECK "YES" OR "NO" FOR EACH OF THE FOLLOWING.] AND [IF "YES", PLEASE INDICATE:]

How much does the condition interfere with your activities? [CHECK THE APPROPRIATE BOX.]

| **NOT ANSWERED**  **(9)** | **NO**  **(0)** | **NOT AT ALL**  **(1)** | **A LITTLE**  **(2)** | **A GREAT DEAL**  **(3)** |  |
| --- | --- | --- | --- | --- | --- |
|  |  |  |  |  | **C8_1** Arthritis or rheumatism |
|  |  |  |  |  | **C8_2** Glaucoma |
|  |  |  |  |  | **C8_3** Asthma |
|  |  |  |  |  | **C8_4** Emphysema or chronic bronchitis |
|  |  |  |  |  | **C8_5** Tuberculosis |
|  |  |  |  |  | **C8_6** High blood pressure |
|  |  |  |  |  | **C8_7** Heart trouble |
|  |  |  |  |  | **C8_8** Circulation trouble in arms or legs |
|  |  |  |  |  | **C8_9** Diabetes |
|  |  |  |  |  | **C8_10** Ulcers (of the digestive system) |
|  |  |  |  |  | **C8_11** Other stomach or intestinal disorders or gall bladder problems |
|  |  |  |  |  | **C8_12** Liver disease |
|  |  |  |  |  | **C8_13** Kidney disease |
|  |  |  |  |  | **C8_14** Other urinary tract disorders (including prostate trouble) |
|  |  |  |  |  | **C8_15** Cancer or Leukemia |
|  |  |  |  |  | **C8_16** Anemia |
|  |  |  |  |  | **C8_17** Effects of stroke |
|  |  |  |  |  | **C8_18** Cerebral Palsy |
|  |  |  |  |  | **C8_19** Parkinson's Disease |
|  |  |  |  |  | C8_20 Multiple Sclerosis |
|  |  |  |  |  | **C8_21** Muscular Dystrophy |
|  |  |  |  |  | **C8_22** Effects of Polio |
|  |  |  |  |  | **C8_23** Epilepsy |
|  |  |  |  |  | **C8_24** Thyroid or other glandular disorders |
|  |  |  |  |  | **C8_25** Skin disorders such as pressure sores, leg ulcers or severe burns |
|  |  |  |  |  | **C8_26** Speech impediment or impairment |
|  |  |  |  |  | **C8_27** Other |

**C9.** Do you have any physical disabilities; such as, total or partial paralysis, missing or non-functional limbs, or broken bones?

___(0) No

___(1) Total paralysis

___(2) Partial paralysis

___(3) Missing or non-functional limbs

___(4) Broken bones

___(9) Not answered

**C10a.** How would you describe your eyesight?

___(1) Excellent

___(2) Good

___(3) Fair

___(4) Poor

___(5) Totally blind

___(9) Not answered

**C10b.** Do you wear glasses or contact lenses to correct vision?

___ (1) Yes

___ (0) No

___(9) Not answered

**C11a.** How is your hearing?

___(1) Excellent

___(2) Good

___(3) Fair

___(4) Poor

___(5) Totally deaf

___(9) Not answered

**C11b.** Do you wear a hearing aid?

___ (1) Yes

___ (0) No

___(9) Not answered

**C12.** Do you have any other physical problems (example, any discomforts with menopausal symptoms) or illnesses at the present time that seriously affect your health?

___(1) Yes

___(0) No

___(9) Not answered

**C12b.** [IF “YES” SPECIFY] ___________________________________________

**C13** Do you use any of the following aids all or most of the time? [CHECK "YES" OR "NO" FOR EACH AID.]

| **NOT ANSWERED**  **(9)** | **YES**  **(1)** | **NO**  **(0)** |  |
| --- | --- | --- | --- |
|  |  |  | **C13_1** Cane (including tripod-trip cane) |
|  |  |  | **C13_2** Walker |
|  |  |  | **C13_3** Wheelchair |
|  |  |  | **C13_4** Leg brace |
|  |  |  | **C13_5** Back brace |
|  |  |  | **C13_6** Artificial limb |
|  |  |  | **C13_7** Hearing aid |
|  |  |  | **C13_8** Colostomy equipment |
|  |  |  | **C13_9** Catheter |
|  |  |  | **C13_10** Kidney dialysis machine or peritoneal dialysis equipment |
|  |  |  | **C13_11** False teeth/ partial or complete dentures |
|  |  |  | **C13_12** Other [SPECIFY]_______________________________________ |

**C14.** Do you need any aids (supportive or prosthetic devices, including false teeth) that you currently do not have?

___(1) Yes

___(0) No

___(9) Not answered

[IF "YES", ASK a.]

**C14b.** What aid do you need? [SPECIFY]_______________________________________

**C15a.** Do you drink alcoholic beverages such as beer, wine, liquors or other spirits?

___(1) Yes

___(0) No (If the answer is NO, go to question C16a)

___(9) Not answered

**C15b.** If you drink beer, on average, about how many cans, bottles or pub/bar glasses* do you drink?

(*One bottle, can, pub/bar glass is = 12 oz)

**C15b1** Quantity (00=none)

**C15b_2** Frequency _______ (1) per day

_______ (2) per week

_______ (9) I do not drink beer or Not Answered

**C15c.** If you drink wine, on average, about how many glasses do you drink?

**C15c_1** Quantity (00=none)

**C15c_2** Frequency _______ (1) per day

_______ (2) per week

_______ (9) I do not drink wine or Not Answered

**C15d.** If you drink liquors and spirits (gin, whisky, vodka, or other mixed drinks), on average, about how many drinks do you have?

**C15d_1** Quantity (00=none)

**C15d_2** Frequency _______ (1) per day

_______ (2) per week

_______ (9) I do not drink liquors and spirits or Not Answered

**C15e**. Has your physician advised you to reduce your alcohol consumption?

___(1) Yes

___(0) No

___(9) Not answered

**C16a.** Do you smoke cigarettes, cigars, a pipe, or chew tobacco?

_____(1) Yes

_____(0) No

_____(9) Not answered

**C16b.** If YES, How many cigarettes, cigars, do you smoke or how many times do you smoke your pipe or chew tobacco per day? ________ NUMBER PER DAY (00=none, 99=Not Answered)

**C17a.** On average, how many hours of sleep do you get each night? _______hrs.

. (Enter using a 24 hour clock, round up to the nearest hour; 99=Not Answered)

**C17a1** hours _____

**C17a2** half-hour ____

**C17b**. What time do you go to bed at night? _____

. (Enter using a 24 hour clock, round up to the nearest hour; 99=Not Answered)

**C17b1** hours _____

**C17b2** half-hour ____

**C17c.** What time do you rise in the morning? _____

. (Enter using a 24 hour clock, round up to the nearest hour; 99=Not Answered)

**C17c1** hours _____

**C17c2** half-hour ____

**C17d.** Do you feel refreshed after a night’s sleep?

___ (1) Yes

___ (0) No

___ (9) Not Answered

**C17e.** Do you have difficulty falling asleep at night?

___ (1) Yes

___ (0) No

___ (9) Not Answered

**C17f.** Do you take naps during the day?

___ (1) Yes

___ (0) No

___ (9) Not Answered

**C18.** Do you regularly participate in any physical activity - such as walking, gardening, hiking, jogging, tennis, biking, or swimming, chopping wood, cleaning house, fetching water, farming, or any activity that is physical in nature? [IF "YES, ASK] HOW OFTEN?

**C18a.** Participate?

___ (1) Yes

___ (0) No

___ (9) Not Answered

**C18b.** How Often?

___(9) Not answered

___(3) Daily

___(2) 3-5 Times per week

___(1) 1-2 Times per week

**C19.** How would you rate your overall health at the present time--excellent, good, fair, or poor?

___(3) Excellent

___(2) Good

___(1) Fair

___(0) Poor

___(9) Not answered

**C20.** Is your health now better, about the same, or worse than it was five years ago?

___(3) Better

___(2) About the same

___(0) Worse

___(9) Not answered

**C21.** How much do your health troubles stand in the way of your doing the things you want to do--not at all, a little (some), or a great deal?

___(3) Not at all

___(2) A little (some)

___(0) A great deal

___(9) Not answered

**FUNCTIONAL ACTIVITIES OF DAILY LIVING**

The next questions are about activities that we all need to do as a part of our daily lives. The questions are asking if you can do these activities without any help at all, OR if you need some help to do them, OR if you can't do them at all without complete help.

BE SURE TO LISTEN ALL ANSWER CHOICES BEFORE YOU SELECT THE ONE THAT DESCRIBES YOU THE BEST.

**INSTRUMENTAL DAILY LIVING TASKS**

**ADL1.** When you have access to a telephone, can you use it...

___(2) without help, including looking up numbers and dialing

___(1) with some help (can answer phone or dial operator in an emergency, but need a special phone or help in getting the number or dialing),

___(0) or are you completely unable to use the telephone?

___(9) Not answered – No access to a telephone

**ADL2.** Can you get to places that are not within walking distance...

___(2) without help (can travel alone on buses, taxis, or drive your own car)

___(1) with some help (need someone to help you or go with you when traveling) or

___(0) are you unable to travel unless emergency arrangements are made for a specialized vehicle like an ambulance?

___(9) Not answered

**ADL3**. Can you go shopping for groceries or clothes…

___(2) without help (taking care of all shopping needs yourself, assuming you have transportation),

___(1) with some help (need someone to go with you on all shopping trips),

___(0) or are you completely unable to do any shopping?

___(9) Not answered

**ADL4**. Can you prepare your own meals?

___(2) without help (plan and cook full meals yourself),

___(1) with some help (can prepare some things but unable to cook full meals yourself),

___(0) or are you completely unable to prepare any meals?

___(9) Not answered

**ADL5**. Can you do your housework...

___(2) without help (can scrub floors, etc.)

___(1) with some help (can do light housework but need help with heavy work)

___(0) or are you completely unable to do any housework?

___(9) Not answered

**ADL6.** Can you [remember to] take your own medicines...

___(2) without help (in the right doses at the right time),

___(1) with some help (able to take medicine if someone prepares it for you and/or reminds you to take it),

___(0) or are you completely unable [to remember to] take your medicines?

___(9) Not answered

**ADL7**. Can you handle your own [financial matters] (money)...

___(2) without help (write checks, pay bills, etc.)

___(1) with some help (manage day-to-day buying but need help with managing your checkbook and paying your bills),

___(0) or are you completely unable to handle [your finances](money)?

___(9) Not answered

**PHYSICAL ACTIVITIES OF DAILY LIVING:**

**ADL8.** Can you eat...

___(2) without help (able to feed yourself completely),

___(1) with some help (need help with cutting, etc.)

___(0) or are you completely unable to feed yourself?

___(9) Not answered

**ADL9**. Can you dress and undress yourself...

___(2) without help (able to pick out clothes, dress and undress yourself)

___(1) with some help

___(0) or are you completely unable to dress and undress yourself?[or are you totally dependent upon someone else to dress and undress you?]

___(9) Not answered

**ADL10.** Can you take care of your own appearance, for example combing your hair and (for men) shaving...

___(2) without help

___(1) with some help,

___(0) or are you completely unable to maintain your appearance yourself?

___(9) Not answered

**ADL11.** Can you walk...

___(2) without help (except for a cane),

___(1) with some help from a person or with the use of a walker or crutches, etc.

___(0) or are you completely unable to walk?

___(9) Not answered

**ADL12.** Can you get in and out of bed...

___(2) without any help or aids

___(1) with some help (either from a person or with the aid of some device)

___(0) or are you totally dependent on someone else to lift you?

___(9) Not answered

**ADL13.** Can you take a bath or shower...

___(2) without help

___(1) with some help (need help getting in and out of the tub, or need special attachments on the tub)

___(0) or are you completely unable to bathe yourself?

___(9) Not answered

**ADL14.** Do you ever have trouble getting to the bathroom on time?

___(2) No

___(0) Yes

___(1) Have a catheter or colostomy

___(9) Not answered

**ADL15.** Is there someone who helps you with such things as shopping, housework, bathing, dressing, and getting around?

[INTERVIEWER NOTE: This question is being asked in reference to functional limitations or losses, and not based on the normal helping that is done as part of interpersonal relationships; e.g., wife helping husband.]

___(1) Yes

___(0) No

___(9) Not answered

[IF "YES" to question 15. PLEASE ANSWER a. AND b.]

**ADL15a.** Who is your primary helper - the person who helps you the most?

___(1) Husband/wife/significant other

___(2) Brother/Sister

___(3) Children

___(4) Grandchildren

___(5) Other relatives

___(6) Friends

___(7) Other

___(9) Not Answered

**ADL15b.** Is there someone else who helps you?

___(1) Husband/wife/significant other

___(2) Brother/Sister

___(3) Children

___(4) Grandchildren

___(5) Other relatives

___(6) Friends

___(7) Other

___(9) Not Answered

**AGING WELL - A STUDY OF ADULT WELL-BEING**

**SECTION D**: MENTAL EFFICACY

This part of the questionnaire asks you some questions about your emotional and mental well-being. There are several parts to the section with each part asking questions about how you feel toward yourself and others.

**I. SELF-ESTEEM**

Please respond to each statement using a five point scale from strongly agree to strongly disagree. [Circle the answer given for each item.]

Strongly Disagree = **S** Disagree = **D** Agree = **A** Strongly Agree = **SA**

Not Answered= **NA** Undecided = **U**

| **SA** | **A** | **U** | **D** | **SD** | **NA** | **Items** |
| --- | --- | --- | --- | --- | --- | --- |
| 1 | 2 | 3 | 4 | 5 | 9 | **DI_1.** I certainly feel useless at times. |
| 1 | 2 | 3 | 4 | 5 | 9 | **DI_2.** On the whole, I am satisfied with myself |
| 1 | 2 | 3 | 4 | 5 | 9 | **DI_3.** I feel I do not have much to be proud of. |
| 1 | 2 | 3 | 4 | 5 | 9 | **DI_4.** I feel that I have a number of good qualities. |
| 1 | 2 | 3 | 4 | 5 | 9 | **DI_5.** At times I think I am no good at all. |
| 1 | 2 | 3 | 4 | 5 | 9 | **DI_6.** I take a positive attitude toward myself. |
| 1 | 2 | 3 | 4 | 5 | 9 | **DI_7.** I wish I could have more respect for myself. |
| 1 | 2 | 3 | 4 | 5 | 9 | **DI_8.** I am able to do things as well as most other people. |
| 1 | 2 | 3 | 4 | 5 | 9 | **DI_9.** All in all, I am inclined to feel that I am a failure |
| 1 | 2 | 3 | 4 | 5 | 9 | **DI_10.** I feel that I'm a person of worth, at least on an equal plane with others. |

**II. SPHERES OF CONTROL**

Please respond to each statement using the seven point scale from strongly agree to strongly disagree. [Circle the answer given for each item.]

Strongly Disagree = **SD** Moderately Disagree = **MD** Disagree = **D**

Strongly Agree = **SA** Moderately Agree = **MA** Agree = **A**

Undecided = **U** Not Answered= **NA**

**A. Perceived Efficacy Section** [Circle the subject's response on each item.]

| **SD** | **MD** | **D** | **U** | **A** | **MA** | **SA** | **NA** | **Perceived Efficacy Statements:** |
| --- | --- | --- | --- | --- | --- | --- | --- | --- |
| 1 | 2 | 3 | 4 | 5 | 6 | 7 | 9 | **DIIA_1**. When I get what I want it’s usually because I worked hard for it. |
| 1 | 2 | 3 | 4 | 5 | 6 | 7 | 9 | **DIIA_2.** When I make plans I am almost certain to make them work. |
| 1 | 2 | 3 | 4 | 5 | 6 | 7 | 9 | **DIIA_3.** I prefer games involving some luck over games requiring pure skill. |
| 1 | 2 | 3 | 4 | 5 | 6 | 7 | 9 | **DIIA_4.** I can learn almost anything if I set my mind to it. |
| 1 | 2 | 3 | 4 | 5 | 6 | 7 | 9 | **DIIA_5.** My major accomplishments are entirely due to my hard work and ability. |
| 1 | 2 | 3 | 4 | 5 | 6 | 7 | 9 | **DIIA_6.** I usually don’t set goals because I have a hard time following through on them. |
| 1 | 2 | 3 | 4 | 5 | 6 | 7 | 9 | **DIIA_7.** Competition discourages excellence. |
| 1 | 2 | 3 | 4 | 5 | 6 | 7 | 9 | **DIIA_8.** Often people get ahead just by being lucky. |
| 1 | 2 | 3 | 4 | 5 | 6 | 7 | 9 | **DIIA_9.** On any sort of exam or competition I like to know how well I do relative to everyone else. |
| 1 | 2 | 3 | 4 | 5 | 6 | 7 | 9 | **DIIA_10.** It’s pointless to keep working on something that’s too difficult for me. |

**B. Interpersonal Control Section [Circle the subject's response on each item.]**

| **SD** | **MD** | **D** | **U** | **A** | **MA** | **SA** | **NA** | **Interpersonal Control Statements:** |
| --- | --- | --- | --- | --- | --- | --- | --- | --- |
| 1 | 2 | 3 | 4 | 5 | 6 | 7 | 9 | **DIIB_1.** Even when I’m feeling self-confident about most things, I still seem to lack the ability to control social situations. |
| 1 | 2 | 3 | 4 | 5 | 6 | 7 | 9 | **DIIB_2.** I have no trouble making and keeping friends. |
| 1 | 2 | 3 | 4 | 5 | 6 | 7 | 9 | **DIIB_3.** I’m not good at guiding the course of a conversation with several others. |
| 1 | 2 | 3 | 4 | 5 | 6 | 7 | 9 | **DIIB_4.** I can usually establish a close personal relationship with someone I find attractive. |
| 1 | 2 | 3 | 4 | 5 | 6 | 7 | 9 | **DIIB_5.** When being interviewed I can usually steer the interviewer toward the topics I want to talk about and away from those I wish to avoid. |
| 1 | 2 | 3 | 4 | 5 | 6 | 7 | 9 | **DIIB_6.** If I need help in carrying off a plan of mine, it’s usually difficult to get others to help. |
| 1 | 2 | 3 | 4 | 5 | 6 | 7 | 9 | **DIIB_7.** If there’s someone I want to meet I can usually arrange it. |
| 1 | 2 | 3 | 4 | 5 | 6 | 7 | 9 | **DIIB_8.** I often find it hard to get my point of view across to others. |
| 1 | 2 | 3 | 4 | 5 | 6 | 7 | 9 | **DIIB_9.** In attempting to smooth over a disagreement I usually make it worse. |
| 1 | 2 | 3 | 4 | 5 | 6 | 7 | 9 | **DIIB_10.** I find it easy to play an important part in most group situations. |

**C. Sociopolitical Control Section [Circle the subject's response on each item.]**

| **SD** | **MD** | **D** | **U** | **A** | **MA** | **SA** | **NA** | **Sociopolitical Control Statements:** |
| --- | --- | --- | --- | --- | --- | --- | --- | --- |
| 1 | 2 | 3 | 4 | 5 | 6 | 7 | 9 | **DIIC_1.** By taking an active part in political and social affairs we, the people, can control world events. |
| 1 | 2 | 3 | 4 | 5 | 6 | 7 | 9 | **DIIC_2.** The average citizen can have an influence on government decisions. |
| 1 | 2 | 3 | 4 | 5 | 6 | 7 | 9 | **DIIC_3.** It is difficult for people to have much control over the things politicians do in office. |
| 1 | 2 | 3 | 4 | 5 | 6 | 7 | 9 | **DIIC_4.** Bad economic conditions are caused by world events that are beyond our control. |
| 1 | 2 | 3 | 4 | 5 | 6 | 7 | 9 | **DIIC_5.** With enough effort we can wipe out political corruption. |
| 1 | 2 | 3 | 4 | 5 | 6 | 7 | 9 | **DIIC_6.** One of the major reasons we have wars is because people don’t take enough interest in politics. |
| 1 | 2 | 3 | 4 | 5 | 6 | 7 | 9 | **DIIC_7.** There is nothing we, as consumers, can do to keep the cost of living from going higher. |
| 1 | 2 | 3 | 4 | 5 | 6 | 7 | 9 | **DIIC_8.** When I look at it carefully I realize it is impossible to have any really important influence over what big businesses do. |
| 1 | 2 | 3 | 4 | 5 | 6 | 7 | 9 | **DIIC_9.** I prefer to concentrate my energy on other things rather than on solving the world’s problems. |
| 1 | 2 | 3 | 4 | 5 | 6 | 7 | 9 | **DIIC_10.** In the long run we, the voters, are responsible for bad government on a national as well as a local level. |

III. Resilience Scale

# Respond to each statement by circling the number that best represents the your feelings toward the statement.

# [Interviewers should complete this set of questions in collaboration with the informant. Show the informant the range of responses (1=Disagree to 7 = Agree) and let the informant tell you which number best represents their feelings toward the statement.]

| **Resilience statements** | **Disagree Agree** | | | | | | | **NA** |
| --- | --- | --- | --- | --- | --- | --- | --- | --- |
| **DIII_1.** When I make plans I follow through with them. | 1 | 2 | 3 | 4 | 5 | 6 | 7 | 9 |
| **DIII_2.** I usually manage one way or another. | 1 | 2 | 3 | 4 | 5 | 6 | 7 | 9 |
| **DIII_3.** I am able to depend on myself more than anyone else. | 1 | 2 | 3 | 4 | 5 | 6 | 7 | 9 |
| **DIII_4**. Keeping interested in things is important to me. | 1 | 2 | 3 | 4 | 5 | 6 | 7 | 9 |
| **DIII_5**. I can be on my own if I have to. | 1 | 2 | 3 | 4 | 5 | 6 | 7 | 9 |
| **DIII_6.** I feel proud that I have accomplished things in my life. | 1 | 2 | 3 | 4 | 5 | 6 | 7 | 9 |
| **DIII_7.** I usually take things in stride. | 1 | 2 | 3 | 4 | 5 | 6 | 7 | 9 |
| **DIII_8.** I am friends with myself. | 1 | 2 | 3 | 4 | 5 | 6 | 7 | 9 |
| **DIII_9.** I feel that I can handle many things at a time. | 1 | 2 | 3 | 4 | 5 | 6 | 7 | 9 |
| **DIII_10.** I am determined. | 1 | 2 | 3 | 4 | 5 | 6 | 7 | 9 |
| **DIII_11.** I seldom wonder what the point of it all is. | 1 | 2 | 3 | 4 | 5 | 6 | 7 | 9 |
| **DIII_12**. I take things one day at a time. | 1 | 2 | 3 | 4 | 5 | 6 | 7 | 9 |
| **DIII_13.** I can get through difficult times because I’ve experienced difficulty before. | 1 | 2 | 3 | 4 | 5 | 6 | 7 | 9 |
| **DIII_14.** I have self-discipline. | 1 | 2 | 3 | 4 | 5 | 6 | 7 | 9 |
| **DIII_15.** I keep interested in things. | 1 | 2 | 3 | 4 | 5 | 6 | 7 | 9 |
| **DIII_16.** I can usually find something to laugh about. | 1 | 2 | 3 | 4 | 5 | 6 | 7 | 9 |
| **DIII_17.** My belief in myself gets me through hard times. | 1 | 2 | 3 | 4 | 5 | 6 | 7 | 9 |
| **DIII_18.** In an emergency, I’m someone people generally can rely on. | 1 | 2 | 3 | 4 | 5 | 6 | 7 | 9 |
| **DIII_19.** I can usually look at a situation in a number of ways. | 1 | 2 | 3 | 4 | 5 | 6 | 7 | 9 |
| **DIII_20.** Sometimes I make myself do things whether I want to or not. | 1 | 2 | 3 | 4 | 5 | 6 | 7 | 9 |
| **DIII_21.** My life has meaning. | 1 | 2 | 3 | 4 | 5 | 6 | 7 | 9 |
| **DIII_22.** I do not dwell on things that I can’t do anything about. | 1 | 2 | 3 | 4 | 5 | 6 | 7 | 9 |
| **DIII_23.** When I’m in a difficult situation, I can usually find my way out of it. | 1 | 2 | 3 | 4 | 5 | 6 | 7 | 9 |
| **DIII_24.** I have enough energy to do what I have to do. | 1 | 2 | 3 | 4 | 5 | 6 | 7 | 9 |
| **DIII_25.** It’s okay if there are people who don’t like me. | 1 | 2 | 3 | 4 | 5 | 6 | 7 | 9 |

**IV. MENTAL STATUS**

**DIV_1.** How often would you say that you worry about things -- very often, fairly often, or hardly ever?

___(0) Very often

___(1) Fairly often

___(2) Hardly ever

___(9) Not answered

**DIV2.** In general, do you find life exciting, pretty routine, or dull?

___(2) Exciting

___(1) Pretty routine

___(0) Dull

___(9) Not answered

**DIV3.** Taking everything into consideration, how would you describe your satisfaction with life in general at the present time -- good, fair, or poor?

___(2) Good

___(1) Fair

___(0) Poor

___(9) Not answered

**DIV4.** Please answer the following question with a Yes or No as they apply to you now. There are no right or wrong answers, only what best applies to you. Occasionally, a question may not seem to apply to you, but please answer either Yes or No, whichever is more nearly correct for you. [CIRCLE “YES” OR “NO” FOR EACH ITEM]

|  | **Yes** | **No** | **Not Answered** |
| --- | --- | --- | --- |
| (1) Do you wake up fresh and rested most mornings? | 1 | 2 | 9 |
| (2) Is your daily life full of things that keep you interested? | 1 | 2 | 9 |
| (3) Have you, at times, very much wanted to leave home? | 1 | 2 | 9 |
| (4) Does it seem that no one understands you? | 1 | 2 | 9 |
| (5) Have you had periods of days, weeks, or months when you couldn't take care of things because you couldn't get going? | 1 | 2 | 9 |
| (6) Is your sleep fitful and disturbed? | 1 | 2 | 9 |
| (7) Are you happy most of the time? | 1 | 2 | 9 |
| (8) Are you being plotted against? | 1 | 2 | 9 |
| (9) Do you feel useless at times? | 1 | 2 | 9 |
| (10) During the past few years, have you been well most of the time? | 1 | 2 | 9 |
| (11) Do you feel weak all over much of the time? | 1 | 2 | 9 |
| (12) Are you troubled by headaches? | 1 | 2 | 9 |
| (13) Have you had difficulty in keeping your balance in walking? | 1 | 2 | 9 |
| (14) Are you troubled by your heart pounding and by a shortness of breath? | 1 | 2 | 9 |
| (15) Even when you are with people, do you feel lonely much of the time? | 1 | 2 | 9 |

**DIV5.** How would you rate your mental or emotional health at the present time -- excellent, good, fair, or poor?

___(3) Excellent

___(2) Good

___(1) Fair

___(0) Poor

___(9) Not answered

**DIV6.** Is your mental or emotional health now - better, about the same, or worse -- than it was five years ago?

___(2) Better

___(1) About the same

___(0) Worse

___(9) Not answered

**AGING WELL - A STUDY OF ADULT WELL-BEING**

SECTION E: LIFE ACTIVITY

This part of the questionnaire asks you some questions about your daily activities including those activities that are considered to be productive as well as leisure activities. The last questions in the section ask you about how you use your time each day.

# I. PRODUCTIVE ACTIVITY INVOLVEMENT

For this part, please list in the appropriate boxes the various **PRODUCTIVE ACTIVITIES** that you are **CURRENTLY DOING OR HAVE DONE IN THE PAST YEAR**. List only those activities that you consider are **PRODUCTIVE ACTIVITIES**. The section that follows this one asks about your **LEISURE OR FREE TIME ACTIVITIES**.

Below are several categories of activities with some examples, please list the activities that you do—they may be different from or the same as the examples that are provided, that’s ok, just list those activities that you do that you think fit under the category. For each activity, please also tell **how frequently you** in the activity using the code key provided in the box below.

## CODE KEY

| How Often do you participate?  0=NOT ANSWERED  NOT SEASONALLY  1 = seldom (couple times per year)   2 = occasionally (about once a month)   3 = frequently (couple times per month)   4 = very often (every week)   5 = daily    SEASONALLY  6 = occasionally (about once a month)   7 = frequently (couple times per month)   8 = very often (every week)   9 = daily |
| --- |

### 1. ACTIVE PARTICIPATION IN

**A. Volunteer Activities**

The first category is **Volunteer Activities.** Some examples of volunteer activities are**:** helping out with home delivered meals, helping out with community services or programs like volunteering at a library or hospital, etc. Check the appropriate box for frequency of participation; if box 6, provide brief explanatory note (e.g., seasonal often, seasonal seldom).

| **List Volunteer Activities-(*)** | **0** | **1** | **2** | **3** | **4** | **5** | **6** | **7** | **8** | **9** |
| --- | --- | --- | --- | --- | --- | --- | --- | --- | --- | --- |
| **EIA_1a**: |  |  |  |  |  |  |  |  |  |  |
| **EIA_2a** |  |  |  |  |  |  |  |  |  |  |
| **EIA_3a** |  |  |  |  |  |  |  |  |  |  |
| **EIA_4a** |  |  |  |  |  |  |  |  |  |  |
| **EIA_5a** |  |  |  |  |  |  |  |  |  |  |
| **EIA_6**: Total number of  "volunteer" activities: |  | | | | | | | | | |

(*) The list of Volunteer activities is given in file ____________

**B. Home Maintenance and Housekeeping**

The second category is **Home Maintenance and Housekeeping**. Examples of home maintenance and housekeeping activities are cleaning house, home redecoration, home repairs, mowing the lawn, etc.

| **List Home Maintenance and Housekeeping Activities-(*)** | **0** | **1** | **2** | **3** | **4** | **5** | **6** | **7** | **8** | **9** |
| --- | --- | --- | --- | --- | --- | --- | --- | --- | --- | --- |
| **EIB_1a**: |  |  |  |  |  |  |  |  |  |  |
| **EIB_2a** |  |  |  |  |  |  |  |  |  |  |
| **EIB_3a** |  |  |  |  |  |  |  |  |  |  |
| **EIB_4a** |  |  |  |  |  |  |  |  |  |  |
| **EIB_5a** |  |  |  |  |  |  |  |  |  |  |
| **EIB_6**: Total number of  " home maintenance  & housekeeping " activities: |  | | | | | | | | | |

(*) The list of Home Maintenance and Housekeeping activities is given in file ____________

**C. Paid Work**

The next productive activity is **Paid Work.** This includes part time work, full time work, contractual work on occasion, etc.

| **List Work Activities- (distinguish between part and full time work)-(*)** | **0** | **1** | **2** | **3** | **4** | **5** | **6** | **7** | **8** | **9** |
| --- | --- | --- | --- | --- | --- | --- | --- | --- | --- | --- |
| **EIC_1a**: |  |  |  |  |  |  |  |  |  |  |
| **EIC_2a** |  |  |  |  |  |  |  |  |  |  |
| **EIC_3a** |  |  |  |  |  |  |  |  |  |  |
| **EIC_4a** |  |  |  |  |  |  |  |  |  |  |
| **EIC_5a** |  |  |  |  |  |  |  |  |  |  |
| **EIC_6**: Total number of  "work" activities: |  | | | | | | | | | |

(*) The list of Home Maintenance and Housekeeping activities is given in file ____________

D. **Assistance to Others**

The last type of productive activity is **Assistance to Others.** Examples include giving direct care to a husband or wife, providing child care assistance, helping friends such as providing transportation or errands, giving home care to a friend, etc.

| List Assistance to Others Activities (*) | **0** | **1** | **2** | **3** | **4** | **5** | **6** | **7** | **8** | **9** |
| --- | --- | --- | --- | --- | --- | --- | --- | --- | --- | --- |
| **EID_1a**: |  |  |  |  |  |  |  |  |  |  |
| **EID_2a** |  |  |  |  |  |  |  |  |  |  |
| **EID_3a** |  |  |  |  |  |  |  |  |  |  |
| **EID_4a** |  |  |  |  |  |  |  |  |  |  |
| **EID_5a** |  |  |  |  |  |  |  |  |  |  |
| **EID_6**: Total number of "assistance to others " activities: |  | | | | | | | | | |

(*) The list of Home Maintenance and Housekeeping activities is given in file ____________

**EI2.** How would you rate your overall satisfaction with your **productive activity** involvement?

Place an “X” in the box that best describes your overall satisfaction.

| highly  dissatisfied  1 | moderately  dissatisfied  2 | undecided/  neither  3 | moderately  satisfied  4 | highly  satisfied  5 | Not Answerd  9 |
| --- | --- | --- | --- | --- | --- |

Are there any activities in the productive activities category that you have reduced your level of involvement over the past year? Please name these activities:

_______________________________ _______________________________

_______________________________ _______________________________

_______________________________ _______________________________

**EI3.** Number of reduced **productive** activities: _________

What are the main reasons for reducing your level of involvement in these activities?

_______________________________ _______________________________

_______________________________ _______________________________

_______________________________ _______________________________

**EI4.** Number of reasons for reducing **productive** activities: _________

#### II. LEISURE ACTIVITY INVOLVEMENT

For this part, please list in the appropriate boxes the various **LEISURE OR FREE TIME ACTIVITIES** that you are **CURRENTLY DOING OR HAVE DONE IN THE PAST YEAR**. List only those activities that you consider are **LEISURE** or **FREE TIME ACTIVITIES**.

In this section, like the previous section, there are several categories of activities with some examples. Please list the activities that you do – they may be different from or the same as the examples that are provided, that’s ok, just list those activities that you do that you think fit under the category. For each activity, please also tell **how frequently you participate** in the activity using the code key provided in the box below.

## CODE KEY

| How Often do you participate?  0=NOT ANSWERED  NOT SEASONALLY  1 = seldom (couple times per year)   2 = occasionally (about once a month)   3 = frequently (couple times per month)   4 = very often (every week)   5 = daily    SEASONALLY  6 = occasionally (about once a month)   7 = frequently (couple times per month)   8 = very often (every week)   9 = daily |
| --- |

### ACTIVE PARTICIPATION IN

#### A. Outdoor Activities

#### The first category is Outdoor Activities. Some examples of outdoor activities are walking, boating, camping, going to the park, bicycling, gardening, nature walks, hunting, etc.

| List Outdoor Activities (*) | **0** | **1** | **2** | **3** | **4** | **5** | **6** | **7** | **8** | **9** |
| --- | --- | --- | --- | --- | --- | --- | --- | --- | --- | --- |
| **EIIA_1a**: |  |  |  |  |  |  |  |  |  |  |
| **EIIA_2a** |  |  |  |  |  |  |  |  |  |  |
| **EIIA_3a** |  |  |  |  |  |  |  |  |  |  |
| **EIIA_4a** |  |  |  |  |  |  |  |  |  |  |
| **EIIA_5a** |  |  |  |  |  |  |  |  |  |  |
| **EIIA_6a** |  |  |  |  |  |  |  |  |  |  |
| **EIIA_7a** |  |  |  |  |  |  |  |  |  |  |
| **EIIA_8**: Total number of " Outdoor " activities: |  | | | | | | | | | |

#### B. Sports

#### Sports is the next category. Examples include football, swimming, jogging, softball league, racquetball, volleyball, soccer, etc.

| **List Sports Activities (*)** | **0** | **1** | **2** | **3** | **4** | **5** | **6** | **7** | **8** | **9** |
| --- | --- | --- | --- | --- | --- | --- | --- | --- | --- | --- |
| **EIIB_1a**: |  |  |  |  |  |  |  |  |  |  |
| **EIIB_2a** |  |  |  |  |  |  |  |  |  |  |
| **EIIB_3a** |  |  |  |  |  |  |  |  |  |  |
| **EIIB_4a** |  |  |  |  |  |  |  |  |  |  |
| **EIIB_5a** |  |  |  |  |  |  |  |  |  |  |
| **EIIB_6a** |  |  |  |  |  |  |  |  |  |  |
| **EIIB_7a** |  |  |  |  |  |  |  |  |  |  |
| **EIIB_8**: Total number of "Sports" activities: |  | | | | | | | | | |

**C. Hobbies and Indoor Activities**

The next category is **Hobbies and Indoor Activities.** Examples of hobbies and indoor activities include baking or cooking for fun, woodworking, needle work, reading, home improvement, painting and art work, crafts, collecting (stamps, coins, etc.), playing a musical instrument, etc.

| **List Hobbies and Indoor Activities (*)** | **0** | **1** | **2** | **3** | **4** | **5** | **6** | **7** | **8** | **9** |
| --- | --- | --- | --- | --- | --- | --- | --- | --- | --- | --- |
| **EIIC_1a**: |  |  |  |  |  |  |  |  |  |  |
| **EIIC_2a** |  |  |  |  |  |  |  |  |  |  |
| **EIIC_3a** |  |  |  |  |  |  |  |  |  |  |
| **EIIC_4a** |  |  |  |  |  |  |  |  |  |  |
| **EIIC_5a** |  |  |  |  |  |  |  |  |  |  |
| **EIIC_6a** |  |  |  |  |  |  |  |  |  |  |
| **EIIC_7a** |  |  |  |  |  |  |  |  |  |  |
| **EIIC_8**: Total number of "Hobbies and Indoor Activities" activities: |  | | | | | | | | | |

#### D. Cultural Activities and Entertainment

#### The fourth category is Cultural Activities and Entertainment. Examples include attending movies, watching TV, listing to the radio, visiting museums, or exhibits, attending plays or concerts, etc.

| **List Cultural and Entertainment Activities (*)** | **0** | **1** | **2** | **3** | **4** | **5** | **6** | **7** | **8** | **9** |
| --- | --- | --- | --- | --- | --- | --- | --- | --- | --- | --- |
| **EIID_1a**: |  |  |  |  |  |  |  |  |  |  |
| **EIID_2a** |  |  |  |  |  |  |  |  |  |  |
| **EIID_3a** |  |  |  |  |  |  |  |  |  |  |
| **EIID_4a** |  |  |  |  |  |  |  |  |  |  |
| **EIID_5a** |  |  |  |  |  |  |  |  |  |  |
| **EIID_6a** |  |  |  |  |  |  |  |  |  |  |
| **EIID_7a** |  |  |  |  |  |  |  |  |  |  |
| **EIID_8**: Total number of " Cultural and Entertainment activities: |  | | | | | | | | | |

##### **E. Home-centered and Social Activities**

##### The next category is **Home-centered and Social Activities.** Examples are playing or spending time with children/grandchildren, taking car trips, entertaining at home, visiting or socializing with friends, telephoning friends or family, socializing with family, eating out, etc.

| **List Home-Centered & Social Activities (*)** | **0** | **1** | **2** | **3** | **4** | **5** | **6** | **7** | **8** | **9** |
| --- | --- | --- | --- | --- | --- | --- | --- | --- | --- | --- |
| **EIIE_1a**: |  |  |  |  |  |  |  |  |  |  |
| **EIIE_2a** |  |  |  |  |  |  |  |  |  |  |
| **EIIE_3a** |  |  |  |  |  |  |  |  |  |  |
| **EIIE_4a** |  |  |  |  |  |  |  |  |  |  |
| **EIIE_5a** |  |  |  |  |  |  |  |  |  |  |
| **EIIE_6a** |  |  |  |  |  |  |  |  |  |  |
| **EIIE_7a** |  |  |  |  |  |  |  |  |  |  |
| **EIIE_8**: Total number of Home-Centered & Social Activities: |  | | | | | | | | | |

F. **Civic Activities**

The next category is **Civic Activities**. Examples are church attendance and socials, community organizations, fraternal groups, auxiliary groups, service organizations, etc.

| **List Civic Activities (*)** | **0** | **1** | **2** | **3** | **4** | **5** | **6** | **7** | **8** | **9** |
| --- | --- | --- | --- | --- | --- | --- | --- | --- | --- | --- |
| **EIIF_1a**: |  |  |  |  |  |  |  |  |  |  |
| **EIIF_2a** |  |  |  |  |  |  |  |  |  |  |
| **EIIF_3a** |  |  |  |  |  |  |  |  |  |  |
| **EIIF_4a** |  |  |  |  |  |  |  |  |  |  |
| **EIIF_5a** |  |  |  |  |  |  |  |  |  |  |
| **EIIF_6a** |  |  |  |  |  |  |  |  |  |  |
| **EIIF_7a** |  |  |  |  |  |  |  |  |  |  |
| **EIIF_8**: Total number of Civic Activities: |  | | | | | | | | | |

**G. Miscellaneous Leisure Activities**

The last category is **Miscellaneous Leisure Activities.** This can include travel or any other activity you consider part of your leisure which doesn’t seem to fit in the above categories.

| **List Miscellaneous Leisure Activities (*)** | **0** | **1** | **2** | **3** | **4** | **5** | **6** | **7** | **8** | **9** |
| --- | --- | --- | --- | --- | --- | --- | --- | --- | --- | --- |
| **EIIG_1a**: |  |  |  |  |  |  |  |  |  |  |
| **EIIG_2a** |  |  |  |  |  |  |  |  |  |  |
| **EIIG_3a** |  |  |  |  |  |  |  |  |  |  |
| **EIIG_4a** |  |  |  |  |  |  |  |  |  |  |
| **EIIG_5a** |  |  |  |  |  |  |  |  |  |  |
| **EIIG_6a** |  |  |  |  |  |  |  |  |  |  |
| **EIIG_7a** |  |  |  |  |  |  |  |  |  |  |
| **EIIG_8**: Total number of Miscellaneous Leisure Activities: |  | | | | | | | | | |

**EII 2.** Compared with five years ago, is your leisure or free activity involvement (check one):

___(1) better

___(2) about the same as before

___(3) worse

___(9) not answered

**EII3.** How would you rate your overall satisfaction with your **leisure or free time activity** involvement?

Place an “X” in the box that best describes your overall satisfaction.

| highly  dissatisfied  1 | moderately  dissatisfied  2 | undecided/  neither  3 | moderately  satisfied  4 | highly  satisfied  5 | Not Answerd  9 |
| --- | --- | --- | --- | --- | --- |

Are there any leisure or free time activities in which you have reduced your participation in the last year? List 1-6 leisure activities in which you have reduced your participation in the last year.

__________________________ __________________________

__________________________ __________________________

__________________________ __________________________

**EII4.** Number of leisure or free time activities __________

**EII5.** Which of the following reasons most closely describe why you have reduced your participation in this/these activity/activities? [Interviewer should read each item as follows: "I am too sick to do these

activities." Place a check mark in the appropriate response column for each item.]

|  | **YES**  **(1)** | **NO**  **(0)** | **NA**  **(9)** | **Reasons for reducing participation in the above activity/activities:** |
| --- | --- | --- | --- | --- |
| **EII5_1** |  |  |  | I am too sick to do this/these activity/activities |
| **EII5_2** |  |  |  | I feel guilty or bad doing this/these activity/activities |
| **EII5_3** |  |  |  | I am too tired to do this/these activity/activities |
| **EII5_4** |  |  |  | I am afraid that others will make fun of me |
| **EII5_5** |  |  |  | I am too old to do this/these activity/activities |
| **EII5_6** |  |  |  | I am afraid of hurting myself |
| **EII5_7** |  |  |  | My family and/or friends do not feel that it is ok to do these activities |
| **EII5_8** |  |  |  | Someone else decides what activities I do |
| **EII5_9** |  |  |  | I am too busy to do this/these activity/activities |
| **EII5_10** |  |  |  | I am not good at this/these activity/activities |
| **EII5_11** |  |  |  | I do not have enough time this/these activity/activities |
| **EII5_12** |  |  |  | I do not have enough money this/these activity/activities |
| **EII5_13** |  |  |  | I only do my leisure/free time activities with friends |
| **EII5_14** |  |  |  | I do not have anyone to teach or instruct me in this/these activity/activities |
| **EII5_15** |  |  |  | I do not have the equipment to do this/these activity/activities |
| **EII5_16** |  |  |  | I do not have a place to do this/these activity/activities |
| **EII5_17** |  |  |  | I do not have anyone with whom to do this/these activity/activities |
| **EII5_18** |  |  |  | I do not know how to do this/these activity/activities |
| **EII5_19** |  |  |  | I am afraid of making a mistake when I do this/these activity/activities |

# III. DAILY TIME DISTRIBUTION

The following questions are about your “non-work, free time” activities and life in general.

## **EIIIA.** Using 24 hours as a total, how many hours a day would you say you spend on:

###### **EIIIA_1 (a,b)** Productive activities (includes housework, paid employment, giving care to others)

###### ______ hours (a) _______ minutes (b)

**EIIIA_2 (a,b)** Personal maintenance (such as sleeping, eating, bathing)

###### ______ hours (a) _______ minutes (b)

**EIIIA_3 (a,b)** Leisure or free time activities

###### ______ hours (a) _______ minutes (b)

## **EIIIB.** Ideally, how much time in a day would you like to spend on:

###### **EIIIB_1 (a,b)** Productive activities (includes housework, paid employment, giving care to others)

###### ______ hours (a) _______ minutes (b)

**EIIIB_2 (a,b)** Personal maintenance (such as sleeping, eating, bathing)

###### ______ hours (a) _______ minutes (b)

**EIIIB_3 (a,b)** Leisure or free time activities

###### ______ hours (a) _______ minutes (b)

**AGING WELL - A STUDY OF ADULT WELL-BEING**

**SECTION F**: MATERIAL SECURITY

This part of the questionnaire asks you some questions about your resources and overall sense of material security. The questions at the end of the section ask you about how satisfied you are with your material resources.

**F1.** Are you presently: [CHECK "YES" OR "NO" FOR EACH OF THE FOLLOWING ALTERNATIVES.]

| **YES**  **(1)** | **NO**  **(0)** | **NA**  **(9)** | **Employment Status:** |
| --- | --- | --- | --- |
|  |  |  | F1_a. Employed full time |
|  |  |  | **F1_b.** Employed part time |
|  |  |  | **F1_c.** Retired |
|  |  |  | **F1_d.** Not employed and seeking work |
|  |  |  | **F1_e**. Not employed and not seeking work |

**F2.** What kind of work have you done most of your life?

_____ (1) Never employed

_____ (2) Housewife or Househusband

_____ (3) OTHER

_____ (9) Not answered

**F2a.** STATE THE SPECIFIC OCCUPATION IN DETAIL: _________________________________________

**F2b.** Coding For Occupation, CIRCLE THE MOST APPROPRIATE ALTERNATIVE

1 = Professional;

2 = Manager or proprietor;

3 = Farmer;

4 = Clerical, sales, or technical;

5 = Skilled, foreman;

6 = Semi-skilled, operative;

7 = Service worker;

8 = Unskilled worker;

9 = Farm laborer.

**F3.** Does your wife/husband work OR did she/he ever work?

[QUESTION APPLIES ONLY TO SPOUSE TO WHOM MARRIED THE LONGEST.]

_____ (1) Yes [IF "YES" ASK F3a. below.]

_____ (2) Never married

_____ (0) No

_____ (9) Not answered

**F3a.** What kind of work did or does she/he do?

STATE THE SPECIFIC OCCUPATION IN DETAIL: ___________________________________________

**F3b.** Coding For Occupation, CIRCLE THE MOST APPROPRIATE ALTERNATIVE

1 = Professional;

2 = Manager or proprietor;

3 = Farmer;

4 = Clerical, sales, or technical;

5 = Skilled, foreman;

6 = Semi-skilled, operative;

7 = Service worker;

8 = Unskilled worker;

9 = Farm laborer.

**F4.** What is your household income per year?**: Enter total amount indicated by respondent:**

**F4_a** (currency)

____(9) Not answered

**F5.** How many people altogether live on this income (that is it provides at least half of their income)?

____ (enter number)

____ (99) Not answered

**F6.** Where does your income (money) come from (you and your wife/husband's)?

|  | **YES (1)** | **NO (0)** | **SOURCE:** |
| --- | --- | --- | --- |
| **F6_1** |  |  | Earnings from employment (wages, salaries, or income from your business) |
| **F6_2** |  |  | Income from rental, interest from investments, etc. (include trusts, annuities, and payments from insurance policies and interest from savings) |
| **F6_3** |  |  | Social Security (include Social Security disability payments but not SSI) |
| **F6_4** |  |  | Veteran's Administration benefits such as GI Bill and disability payments |
| **F6_5** |  |  | Disability payments not covered by Social Security, SSI, or VA. Both government and private, and including Workman's Compensation |
| **F6_6** |  |  | Unemployment compensation |
| **F6_7** |  |  | Retirement pension from job |
| **F6_8** |  |  | Alimony or child support |
| **F6_9** |  |  | Scholarships, stipends (include only the amount beyond tuition) |
| **F6_10** |  |  | Regular financial assistance from family members (include regular contributions from employed children) |
| **F6_11** |  |  | SSI payments (yellow government checks) |
| **F6_12** |  |  | Regular financial aid from private organizations and churches |
| **F6_13** |  |  | Welfare payments and Aid to Dependent Children |
| **F6_14** |  |  | Social assistance |
| **F6_15** |  |  | Other: Specify source(s): _______________________________________ |

[When finished with responses in table, GO BACK TO Q. 4. and ask respondent to verify total annual income]

**F7.** The following questions are about your home. Do you own your own home?

_____ (1) Yes

_____ (0) No

_____ (9) Not answered

**F7a.** If you own your home, do you own your home outright or are you still paying a mortgage?

_____ (1) Own outright

_____ (2) Still paying mortgage

_____ (9) Not answered

**F7b.** If you DO NOT own your home, do you (and your wife/husband) pay the total rent for your home (house or apartment) or do you contribute to the cost, or does someone else own it or pay the rent?

_____ (1) Subject pays total rent

_____ (2) Subject contributes to the cost

_____ (3) Someone else owns it or pays the rent (subject does not contribute)

_____ (9) Not answered

**F7c.** Do you live in public housing or receive a rent subsidy?

_____ (0) No, neither

_____ (1) Yes, live in public housing

_____ (2) Yes, receive a rent subsidy

_____ (9) Not answered

**F8a.** Do you feel that you have a decent place in which to live?

_____ (1) Yes

_____ (0) No

_____ (9) Not answered

**F8b.** Do you feel safe living here?

_____ (1) Yes

_____ (0) No

_____ (9) Not answered

**F9.** Are your assets and financial resources sufficient to meet emergencies?

_____ (1) Yes

_____ (0) No

_____ (9) Not answered

**F10.** Are your expenses so heavy that you cannot meet the payments, or can you barely meet the payments, or are your payments no problem to you?

______ (1) I cannot meet my payments

______ (2) I can barely meet payments

______ (3) Payments are no problem

______ (9) Not answered

**F11.** Is your financial situation such that you feel you need financial assistance or help beyond what you are already getting?

_____ (1) Yes

_____ (0) No

_____ (9) Not answered

**F12a.** Do you pay for your own food

_____ (1) Yes

_____ (0) No

_____ (9) Not answered

**F12b.** If YES: Do you feel that you need help in paying for and obtaining your food?

_____ (1) Yes

_____ (0) No

_____ (9) Not answered

**F12c.**  If **F12b** is YES, ask: From where do you get help with food cost and meals?

| **YES**  **(1)** | **NO**  **(0)** | **NA**  **(9)** | **SOURCE:** |
| --- | --- | --- | --- |
|  |  |  | F12c_1. Family or friends |
|  |  |  | **F12c_2.** Food stamps or government aid |
|  |  |  | **F12c_3.** Prepared food (meals) from an agency or organization program.  **F12c_4. Specify the number of meals per week ______** |

**F13.** Are you covered by any kind of health care program or medical insurance (public or private)?

_____ (1) Yes [IF "yes" ASK a. below.]

_____ (0) No

_____ (9) Not answered

**F13a.** What kind?

| **YES**  **(1)** | **NO**  **(0)** | **NA**  **(9)** | **SOURCE:** |
| --- | --- | --- | --- |
|  |  |  | **F13a_1.** National Health Care Program (e.g., Medicaid in the USA) |
|  |  |  | **F13a_2.** National Hospitalization Program (e.g., in the USA, Medicare Plan A only - hospitalization only) |
|  |  |  | **F13a_3.** National Health Insurance (e.g., in the USA, Medicare Plans A and B – for hospitalization and doctors' bills) |
|  |  |  | **F13a_4.** Other insurance: hospitalization only (Blue Cross or other) |
|  |  |  | **F13a_5.** Other insurance: hospitalization and doctors' bills (major medical or other |

**F13b.** Are you able to pay for your medications, eye glasses, dentures, prosthetics, dialysis, or visits to the doctor, dentist, eye care specialist, etc.?

____ (1) Yes

____ (0) No

____ (9) Not answered

**F13c_1**. How far must you travel to reach the nearest doctor or hospital? (999=Not Answered)

**F13c_2**. 1=miles/ 2=kilometer (Circle scale)

**F14.** As compared to other people your age, please tell me how well you think you are **now** doing financially -- better, about the same, or worse?

_____ (2) Better

_____ (1) About the same

_____ (0) Worse

_____ (9) Not answered

**F15.** How well do you feel your needs are met by the financial resources you have (e.g. money) -- very well, fairly well, or poorly?

_____ (2) Very well

_____ (1) Fairly well

_____ (0) Poorly

_____ (9) Not answered

**F16.** Do you usually have enough money to buy or obtain small luxuries, or those little "extras"?

_____ (2) Yes

_____ (0) No

_____ (9) Not answered

**F17.** At the present time, do you feel that you will have enough money for your needs in the future?

_____ (2) Yes

_____ (0) No

_____ (9) Not answered

**AGING WELL - A STUDY OF ADULT WELL-BEING**

**SECTION G:** PERCEPTIONS OF AGING WELL

This part of the questionnaire asks about your satisfaction with life. Below are some statements about life in general. Would you please respond to each item by indicating whether you **AGREE** or **DISAGREE** with the statement by circling the number below the correct column. If you are not sure one way or the other, please indicate that you are **UNSURE OR DO NOT KNOW HOW YOU FEEL.** In order for the researchers to use this scale, a response to every item is needed; please do not leave any question unanswered.

| **STATEMENTS:** | **AGREE** | **DIS-AGREE** | **NOT SURE?** |
| --- | --- | --- | --- |
| **G_1**. As I grow older, things seem better than I thought they would be. | 2 | 0 | 1 |
| **G_2.** I have gotten more of the breaks in life than most of the people I know. | 2 | 0 | 1 |
| **G_3.** This is the dreariest time of my life. | 0 | 2 | 1 |
| **G_4.** I am just as happy as when I was younger. | 2 | 0 | 1 |
| **G_5.** These are the best years of my life. | 2 | 0 | 1 |
| **G_6.** Most of the things I do are boring or monotonous. | 0 | 2 | 1 |
| **G_7**. The things I do are as interesting to me as they ever were. | 2 | 0 | 1 |
| **G_8**. As I look back on my life, I am fairly well satisfied. | 2 | 0 | 1 |
| **G_9.** I have made plans for things I'll be doing a month or a year from now. | 2 | 0 | 1 |
| **G_10**. When I think back over my life, I didn't get most of the important things I wanted | 0 | 2 | 1 |
| **G_11.** Compared to other people, I get down in the dumps too often. | 0 | 2 | 1 |
| **G_12**. I've gotten pretty much what I expected out of life. | 2 | 0 | 1 |
| **G_13.** In spite of what people say, the lot of the average person is getting worse, not better. | 0 | 2 | 1 |

**G14.** Below is a ladder on which you are asked to rate your present life in relation to your ideas about the *worst possible* and *best possible life*. The top rung of the ladder represents the *best possible life* as you think of it and the bottom rung represents the *worst possible life* as you would think of it. Please place an "**X**" **on the rung** that best represents your current situation. **Be sure to place your “X” on the rung or line. (99=Not Answer)**

| **Best Possible Life=10** |
| --- |
| 9 |
| 8 |
| 7 |
| 6 |
| 5 |
| 4 |
| 3 |
| 2 |
| 1 |
| **Worse Possible Life =0** |

Source:

The lists of activities are compatible with the lists of activities made by the Dutch National Social and Cultural Planning Board for the time budget sets of data and data sets on social participation.

# SECTION E: LIFE ACTIVITY

# E I 1.A Volunteer activities

Do you participate in volunteer work? For example being member of a board, organisational work, administrative work, visiting, transporting, caring for persons, house-to-house collection, coaching. Which kind of organisations do you work for?

List:

1. Hospitals, homes for the elderly, nursing homes
2. Community centres, neighbourhood centres, committees of residents
3. Schools, day care centres, youth work
4. Sports and recreation
5. Cultural organisations
6. Libraries
7. Trade Unions, professional organisations
8. Political parties
9. Action committees, organisations for development issues, environmental issues, peace, human rights, nature, animal protection
10. Churches, religious organisations
11. Women’s organisations, elderly organisations
12. other

# E I 1.B Home maintenance and house keeping

Do you participate in home maintenance and housekeeping?

List:

1. Preparing meals (cooking, making coffee, dishwashing, setting the table)
2. Cleaning (cleaning, vacuuming, dusting, scrubbing, window cleaning, car washing)
3. Tidying up
4. Washing, ironing, repairing clothes
5. Child care
6. Gardening, watering the plants
7. Pets care, taking the dog out
8. Shopping, take-away, getting petrol, visiting bank or post office
9. Home maintenance and repairs
10. Administration (paying bills, filling forms, writing letters)
11. other

# E I 1.C Paid work

Do you participate in paid work? How many jobs or functions do you have? Are these part-time or full-time?

(no list)

# E I 1.D Assistance to others

Do you help other persons (direct care to a partner, friends, relatives)? What kind of help do you give?

List:

1. Personal care
2. Housekeeping
3. Child care
4. Home maintenance
5. Transport
6. Administration
7. Visiting elderly or disabled persons
8. Other

# II LEISURE ACTIVITY INVOLVEMENT

**E II 1.A Outdoor activities**

Do you participate in outdoor activities?

List

1. Walking
2. Making cycling tours
3. Boating
4. Going to a park, wood or nature area
5. Fishing
6. Gardening as a hobby (own garden or community garden)
7. Going to a camp site, recreation home or beach house)
8. Other

# E II 1.B Sports

Do you participate in sports?

(no list)

# E II 1.C Hobbies

Do you participate in hobbies at home?

List:

1. Cooking or baking as hobby
2. Knitting, embroidering, crochet, sewing as hobby
3. Puzzling, jigsawing
4. Playing a music instrument, singing
5. Painting, drawing, modelling, pottery making
6. Handicraft
7. Collecting (stamps, coins)
8. Other

# E II 1.D Cultural activities, entertainment and media

Do you participate in cultural activities, entertainment and media?

List:

1. Going to the movies
2. Visiting a concert or theatre
3. Visiting a museum or exhibition
4. Visiting a sports match
5. Visiting an event/ fair
6. Watching TV, listening to the radio
7. Reading newspapers, magazines, journals
8. Internet surfing
9. Other

# E II 1.E Home-centred and social activities

Do you participate in home-centred and social activities?

List:

1. Visiting
2. Receiving visitors
3. Day trips with partner, children, grandchildren, relatives, friends, acquaintances
4. Eating out
5. Going to a pub
6. Phoning, chatting, emailing
7. Other

# E II 1.F Civic activities

Do you join civic activities (not volunteer **work**)?

List:

1. Community centres, neighbourhood centres, committees of residents
2. Sports and recreational clubs
3. Cultural associations and organisations
4. Trade Unions, professional organisations
5. Political parties
6. Action committees, organisations for development issues, environmental issues, peace, human rights, nature, animal protection
7. Churches, religious organisations
8. Women’s organisations, elderly organisations
9. Other

# E II 1.G Other leisure activities

Do you participate in other leisure activities for example travelling or other activities not mentioned?

(no list)
